# Association between genome-wide epigenetic and genetic alterations in breast cancer tissue and response to HER2-targeted therapies in HER2-positive breast cancer patients: new findings and a systematic review

**DOI:** 10.20517/cdr.2022.63

**Published:** 2022-11-02

**Authors:** Daniela Furrer, Dzevka Dragic, Sue-Ling Chang, Frédéric Fournier, Arnaud Droit, Simon Jacob, Caroline Diorio

**Affiliations:** ^1^Centre de Recherche sur le cancer de l’Université Laval, 1050 Avenue de la Médecine, Québec, QC G1V 0A6, Canada.; ^2^Axe Oncologie, Centre de Recherche du CHU de Québec-Université Laval, Québec, QC G1S 4L8, Canada.; ^3^Département de médecine sociale et préventive, Faculté de Médecine, Université Laval, Québec, QC G1V 0A6, Canada.; ^4^Université Paris-Saclay, UVSQ, Inserm, CESP U1018, Exposome and Heredity Team, Gustave Roussy, Villejuif 94807, France.; ^5^Département de médecine moléculaire, Faculté de Médecine, Université Laval, Québec, QC G1V 0A6, Canada.; ^6^Département de biologie moléculaire, de biochimie médicale et de pathologie, Faculté de Médecine, Université Laval, Québec, QC G1V 0A6, Canada.; ^7^Centre des Maladies du Sein, Hôpital du Saint-Sacrement, Québec, QC G1S 4L8, Canada.

**Keywords:** Breast neoplasms, epigenetics, genetics, HER2 inhibitors, treatment response, biomarkers

## Abstract

Recent evidence suggests that genetic and epigenetic mechanisms might be associated with acquired resistance to cancer therapies. The aim of this study was to assess the association of genome-wide genetic and epigenetic alterations with the response to anti-HER2 agents in HER2-positive breast cancer patients. PubMed was screened for articles published until March 2021 on observational studies investigating the association of genome-wide genetic and epigenetic alterations, measured in breast cancer tissues or blood, with the response to targeted treatment in HER2-positive breast cancer patients. Sixteen studies were included in the review along with ours, in which we compared the genome-wide DNA methylation pattern in breast tumor tissues of patients who acquired resistance to treatment (case group, *n *= 6) to that of patients who did not develop resistance (control group, *n *= 6). Among genes identified as differentially methylated between the breast cancer tissue of cases and controls, one of them, *PRKACA*, was also reported as differentially expressed in two studies included in the review. Although included studies were heterogeneous in terms of methodology and study population, our review suggests that genes of the PI3K pathway may play an important role in developing resistance to anti-HER2 agents in breast cancer patients. Genome-wide genetic and epigenetic alterations measured in breast cancer tissue or blood might be promising markers of resistance to anti-HER2 agents in HER2-positive breast cancer patients. Further studies are needed to confirm these data.

## INTRODUCTION

The human epidermal growth factor receptor 2 (HER2) is a transmembrane tyrosine kinase receptor and belongs to the epidermal growth factor receptor (EGFR) family^[1]^. It comprises an extracellular domain (ECD), a transmembrane segment, and an intracellular region^[2]^. *HER2* gene amplification and receptor overexpression, which occur in approximately 15%-20% of breast cancer patients, are important markers for poor prognosis, including a more aggressive disease and shorter survival^[3]^. In addition, HER2-positive status is considered a predictive marker of response to HER2-targeted drugs^[4]^. Detection of receptor overexpression via immunohistochemistry (IHC) and/or *HER2* gene amplification using *in situ* hybridization (ISH) techniques in breast cancer tissue, including fluorescent ISH (FISH), determines patients’ eligibility to receive anti-HER2 therapies^[5]^. Food and Drug Administration (FDA)-approved anti-HER2 agents currently used in clinical settings in combination with chemotherapy comprises recombinant monoclonal antibodies that bind the ECD of HER2, such as trastuzumab (Herceptin®), pertuzumab (Perjeta®) and trastuzumab emtansine (T-DM1, Kadcyla®), and small molecule tyrosine kinase inhibitors, like lapatinib (TYKERB®), that inhibit enzyme function of the intracellular catalytic domain of HER2 and other EGFR members^[6,7]^.

Although targeted treatment with anti-HER2 agents has significantly improved the disease-free and overall survival rates of metastatic and early-stage HER2-positive breast cancer patients^[8-11]^, resistance to anti-HER2 therapy, both primary and acquired, has emerged as a major clinical problem in the treatment of HER2-positive breast cancer patients^[12-14]^. Even though several molecular mechanisms of resistance to anti-HER2 agents have been proposed in preclinical models, no clinically applicable strategy to overcome resistance to these targeted treatments has been identified yet^[15]^. Therefore, there is an urgent need to identify reliable predictive molecular markers of treatment failure with the ultimate goal of developing targeted drugs that can overcome resistance.

Studies indicate that genetic factors, including single nucleotide polymorphisms (SNPs), copy number variations (CNVs), *HER2* mutations, and HER2 splice variants, might influence treatment effectiveness toward targeted therapies in HER2-positive breast cancer patients or HER2-positive breast cancer cell lines^[16-28]^. Recent evidence suggests that epigenetic regulatory mechanisms, including DNA methylation and microRNAs (miRNAs), might play a role in acquiring resistance to cancer therapies^[29-32]^. DNA methylation occurs through the covalent attachment of a methyl group on cytosine residues in CpG dinucleotides and contributes to transcriptional regulations^[33]^. While DNA methylation in the immediate vicinity of the transcriptional start site (TSS) generally represses gene expression, methylation in the gene body (far from annotated TSS) may stimulate elongation and is, therefore, positively associated with gene expression^[34,35]^. miRNA are approximately 22 nucleotides long non-coding RNAs that regulate gene expression in a sequence-specific manner^[36]^.

The aim of this pilot study was to analyze the association between DNA methylation patterns in breast cancer specimens and response to trastuzumab in a cohort of 12 trastuzumab-treated, non-metastatic HER2-positive breast cancer patients. Additionally, a systematic review that combines these findings with all available published results on the association of genome-wide genetic and epigenetic alterations in breast cancer tissue or blood with response to anti-HER2 treatment in HER2-positive breast cancer patients is also reported.

## MATERIAL AND METHODS

### Pilot study of new findings

#### Study population and data collection

The study population consisted of 12 women (six cases and six controls) selected among 106 trastuzumab-treated patients with non-metastatic, HER2-positive breast cancer diagnosed between July 1, 2005 and December 31, 2010 at the Centre des Maladies du Sein, a specialized breast center in Quebec City, Canada. Information on tumor characteristics and prognostic factors at the time of diagnosis (baseline) and follow-up information were collected from medical records. The clinical endpoint in this study was disease-free survival (DFS). All breast cancer recurrences (locoregional, contralateral breast, and distant) were considered as events, whereas death (from any cause) before recurrence and loss to follow-up were considered as censoring events. 

Over a mean follow-up period of 6.22 years, 22 patients out of 106 experienced recurrence. Eight cases were randomly selected among all patients who developed recurrence during follow-up and six had a sufficient amount of primary breast cancer tissue available for DNA extraction (see below). Of note, baseline characteristics of the six selected cases were comparable to the total population of cases (*n *= 22) for all characteristics except tumor grade (the proportion of grade III tumors among the selected cases was 67% *vs.* 41% for the total population of cases) [Supplementary Table 1]. For each case, one control was selected from the 84 patients who had not developed recurrence and were alive at the date of the case’s recurrence. Controls were matched to cases for the following factors: age at diagnosis (with 5-year age categories), estrogen receptor (ER) status, year of diagnosis (with 2-year categories), and menopausal status. The number of samples used was determined upon the availability of samples and not evaluated using a statistical sample size calculation. All patients provided written informed consent. Ethical approval of the study was obtained from the Research Ethics Committee of the Centre de Recherche du CHU de Québec (# 2016-2802).

#### Gene methylation assessment

To ensure that DNA methylation was analyzed to the greatest possible extent in breast cancer tissue and to reduce contamination with other cell types (lymphocytes, adipocytes, fibroblasts), tissue microarray (TMA) blocks containing formalin-fixed, paraffin-embedded (FFPE) breast cancer tissue cores (1 mm in diameter) were constructed for each patient, as previously described^[37]^. From each TMA block, one section was stained with hematoxylin and eosin (H&E) to verify the cellular composition of the cores. Cores were removed from TMA blocks if they contained abnormal tissue or if epithelial tumor tissue occupied < 70% of the core area before proceeding to DNA extraction. H&E sections were prepared from different levels of the TMA blocks: at the beginning, at regular intervals (every tenth 10-µm-thick serial section), and after the last section. DNA was extracted from tissue cores using GeneJET FFPE DNA Purification kit (ThermoScientific, Ottawa, Canada) with minor modifications to the manufacturer’s instructions in which samples were incubated with Digestion buffer for six minutes and incubated with Proteinase K solution for 180 minutes. 

DNA samples were sent to Génome Québec Innovation Center (Montreal, Canada). Methylation was measured with the Illumina HumanMethylation450 BeadChip array (Illumina Inc., San Diego, CA, USA) following the manufacturer’s instructions for the bisulfite treatment, Infinium FFPE quality control (Illumina FFPE QC kit, Illumina, Inc., CA, USA), and DNA restoration. This BeadChip interrogates 482,421 CpG sites, 3091 non-CpG sites, 65 random SNPs, and covers 21,231 RefSeq genes. It uses two distinct oligonucleotide probes (Infinium I and Infinium II) to assess methylation levels^[38]^.

#### Statistical analysis

Raw β-values, defined as the ratio of the methylated probe intensity to the overall intensity (sum of the methylated and unmethylated probe intensities)^[39]^, were imported into the R statistical programming environment (version 3.2.2). Since M-values [logit transformed β-values, calculated as 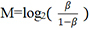] are considered more reliable in the detection rate and true positive rate for both highly methylated and unmethylated CpG sites compared to β-values^[39]^, M-values were used for statistical analyses.

Quality control was performed with the qcReport function from the minfi package, and none of the 12 samples were excluded due to bad quality control. The Dasen method from the WateRmelon package, also known as data-driven separate normalization, was used to background correct and quantile normalize data based on methylated and unmethylated intensities, separately, by probe types (Infinium I and II)^[40]^. A probe filtration step was performed to remove CpG sites corresponding to probes that could affect our analysis, including probes with bad detection (detection *P*-value > 0.01); unique probes having a common single nucleotide polymorphism (SNP) in European individuals at the interrogated CpG loci or the single-base extension according to the list published by Chen *et al.*^[41]^; probes that can hybridize to multiple loci also listed by Chen *et al.*^[41]^; and probes located on X and Y chromosomes. A total of 76,161 unique probes were removed, leaving 406,260 autosomal probes for the analysis. Data were verified for confounding batch effects due to separate chips^[42]^, and none were observed. All samples passed quality-control tests and were therefore retained in the analysis.

Baseline characteristics between cases and controls were compared using Fisher’s exact test for categorical variables, Student’s *t*-test for follow-up time and Wilcoxon-Mann-Whitney test for the other continuous variables. The difference in global methylation levels between median M-values of cases and controls was assessed using a Wilcoxon signed-rank test for paired samples. Differentially methylated probes (DMPs) were identified using LIMMA (robust linear regression method), taking into account the matching factors between cases and controls (i.e., age at diagnosis, ER status, year of diagnosis, and menopausal status). Multiple testing correction was performed using false discovery rate (FDR) estimation (cut-off < 5%). In addition, we used a log_2_-fold change |log2FC| (i.e., the difference between mean M-values measured in breast cancer tissues of resistant patients and controls) > 2.0 as a cut-off to identify probes that were strongly differentially methylated between cases and controls. 

### Systematic review of published findings

A systematic review was conducted and reported according to the 2020 Preferred Reporting Items for Systematic Reviews and Meta-Analyses (PRISMA) guidelines^[43]^.

#### Eligibility criteria

*Population:* We included studies of HER2-positive breast cancer patients treated with any type of anti-HER2 agent (trastuzumab [Herceptin®], lapatinib [TYKERB®], pertuzumab [Perjeta®], trastuzumab emtansine [trastuzumab-DM1, Kadcyla®], erlotinib [Traceva®], gefitinib [Iressa®]) regardless of age, stage, and menopausal status.

*Exposure:* To be included, a study had to measure response-specific survival using including pathologic complete response (pCR), disease-free survival (DFS), progression-free survival (PFS), or event-free survival (EFS).

*Outcome:* We considered all assessments of genetic and epigenetic alterations at the genome-wide level in breast cancer tissue or blood, whatever the measurement method.

*Types of studies*: Any observational or randomized controlled study that assessed the association between genome-wide genetic and epigenetic alterations in breast cancer specimens or blood and the response to anti-HER2 agents in HER2-positive breast cancer patients was eligible. Case reports were excluded.

Only full available articles in English were included.

#### Information sources

Dragic D searched the PubMed biomedical database from inception to the search date of March 25, 2021, to identify eligible studies.

#### Search strategy

The search strategy was developed by Dragic D and Furrer D, approved by Diorio C, using controlled vocabulary search terms and free-text words related to HER2-positive breast cancer, genome-wide genetic and epigenetic alterations and treatment outcome [Supplementary Table 2]. No restrictions regarding language were applied. 

#### Selection process

The references identified by the search strategy were selected according to the predefined eligibility criteria in a two-step process: titles and abstracts were screened by one author (Dragic D), and full texts of retained articles were examined by two authors (Dragic D and Furrer D). Disagreements between the two authors were discussed until a consensus was reached, and whenever required, a third review author (Diorio C) was consulted. 

#### Data collection process

We designed a data extraction form for this review. Data were extracted by two authors (Furrer D and Dragic D) for half of the included studies. A third author (Diorio C) was consulted when discrepancies between both authors could not be resolved. For the remaining studies, the extraction was done by one author (Dragic D), and when needed, a second author (Diorio C) was involved. Additionally, the authors of studies of interest that lacked data to evaluate eligibility (*n *= 1) or other measures needed for this review (*n *= 3) were contacted to obtain the necessary information.

#### Data items

For all selected articles, study characteristics (study design and sample size), patient’s characteristics (age, stage, ER and PR status, menopausal status, and treatment received), assessment of genetic and epigenetic alterations (tissue processing, DNA, RNA or miRNA extraction method, assessment method, and parameters used), as well as statistical methods and study results, were collected. The study’s definition of response to targeted treatment was recorded.

#### Risk of bias assessment

Studies included in the review were assessed for risk of bias using the “Risk Of Bias in Non-randomized Studies of Interventions (ROBINS-I) tool^[44]^. The following domains were assessed: selection of participants in the study, exposure measurement, outcome measurement, potential confounding factors, missing data, and selective reporting.

Assessment of the risk of bias was done by two authors (Furrer D and Dragic D) for half of the included studies. Inconsistencies were discussed to reach a consensus. For the remaining studies, the assessment was done by one author (Dragic D), and when required, a second reviewer (Furrer D) was consulted.

#### Assessment of heterogeneity

Differences between studies, including study design, patient characteristics (age, menopausal status, ethnicity, and treatment received), tumor characteristics (stage, ER status), assessment of genome-wide genetic and epigenetic alterations (tissue processing, extraction method, and measurement method), and different levels of risk of bias were considered for exploring possible sources of heterogeneity.

#### Synthesis methods

Considering that high heterogeneity between studies was expected, quantitative data synthesis was not considered appropriate. Instead, we adopted a qualitative systematic review approach to investigate the relationship between epigenetic and genetic alterations and response to HER2-targeted therapies in HER2-positive breast cancer patients. The selection process was detailed using the PRISMA 2020 flow diagram. Extracted data were first reported in a table gathering summary characteristics of all included studies. Additional information specific to the epigenetic or genetic method used was detailed in several tables. Results and genes identified by several studies were also highlighted in a table. If pathway analysis was not presented, we performed pathway analysis using the list of the differentially expressed genes reported by the study authors and the PANTHER online software (Protein Analysis Through Evolutionary Relationships). *P*-values < 0.05 were considered significant.

## RESULTS

### Pilot study of new findings

Genome-wide DNA methylation data in 12 breast cancer specimens were obtained from six trastuzumab-treated HER2-positive breast cancer patients who experienced recurrence during follow-up (cases) and six individually matched patients who had not developed recurrence and were alive at the date of the case’s recurrence (controls). Baseline characteristics of cases and controls are summarized in Table 1. Baseline characteristics for both groups were comparable in clinicopathological characteristics (tumor grade, lymph node status, and tumor size) and treatment received. Compared to controls, a higher proportion of cases (50%) had a body mass index > 25 kg/m^2^, although not statistically significant. 

**Table 1:** Baseline characteristics of the whole study population, case group and control group

**Factor**	**Whole study (*n* = 12)**	**Cases (*n* = 6)**	**Controls (*n* = 6)**	***P*** **-value**
	Mean ± SD	Mean ± SD	Mean ± SD	
Age (years)	51.3 ± 5.6	51.3 ± 6.1	51.2 ± 5.6	0.87
Body mass index (kg/m^2^)	23.8 ± 2.2	23.8 ± 2.6	23.9 ± 1.6	0.94
Follow-up time (years)	5.5 ± 2.9	3.8 ± 2.8	7.2 ± 2.1	0.04
	*n* (%)	*n* (%)	*n* (%)	
Grade I/II III	3 (23%) 9 (77%)	2 (33%) 4 (67%)	1 (17%) 5 (83%)	1.00
Lymph node status Negative Positive	1 (8%) 11 (92%)	0 (0%) 6 (100%)	1 (17%) 5 (83%)	1.00
Tumor size (cm) ≤ 5 > 5	11 (92%) 1 (8%)	5 (83%) 1 (17%)	6 (100%) 0 (0%)	1.00
Estrogen receptor status Negative Positive	4 (33%) 8 (67%)	2 (33%) 4 (67%)	2 (33%) 4 (67%)	1.00
Progesterone receptor status Negative Positive	6 (50%) 6 (50%)	3 (50%) 3 (50%)	3 (50%) 3 (50%)	1.00
Menopausal status Pre Post	4 (33%) 8 (67%)	2 (33%) 4 (67%)	2 (33%) 4 (67%)	1.00
Radiotherapy No Yes	1 (8%) 11 (92%)	1 (17%) 5 (83%)	0 (0%) 6 (100%)	1.00
Endocrine therapy No Yes	4 (33%) 8 (67%)	2 (33%) 4 (67%)	2 (33%) 4 (67%)	1.00
Chemotherapy No Yes	0 (0%) 12 (100%)	0 (0%) 6 (100%)	0 (0%) 6 (100%)	1.00
Trastuzumab treatment completed No Yes	0 (0%) 12 (100%)	0 (0%) 6 (100%)	0 (0%) 6 (100%)	1.00

*n*: Number of subjects; SD: standard deviation.

Global methylation levels between cases and controls were not statistically different: the median M-values of cases were 0.487, and the median M-values of controls were 0.504 (*P*-value: 0.844). At probe methylation levels, we identified 2,009 CpGs (1,382 genes) that were differentially methylated between cases and controls: 1,200 DMPs (885 genes) were significantly hypermethylated and 809 DMPs (497 genes) were significantly hypomethylated in tumor tissues of cases compared to those of controls after multiple testing correction (FDR < 0.05). 

Fifteen genes had a |log2FC| > 2.0: ten genes (*SIX2*, *PLEC1*, *ZNF833*, *RAI1*, *ZNF598*, *USP4*,* DOCK1*, *UNC84A*, *KLF16*, *PRKACA*) were significantly hypermethylated, and five genes [*STK33*, *TBXT *(alias* T*), *KCNH7*, *ADAMTS2*, *FAM19A5*] were significantly hypomethylated in breast cancer tissues of cases compared to controls. Results are reported in Table 2.

**Table 2:** Genes differentially methylated in breast cancer tissues of cases compared to controls

**CpG sites**	**Chr**	**Gene**	**Gene region**	**CpG island region**	**LogFC**	**q-value**
cg08788717	chr11	STK33	TSS200	Island	-2.835	0.036
cg02149708	chr6	TBXT (T)	TSS200	Island	-2.384	0.038
cg26974327	chr2	KCNH7	TSS200	OpenSea	-2.150	0.037
cg05214690	chr5	ADAMTS2	1stExon	Island	-2.127	0.015
cg22643811	chr22	FAM19A5 (TAFA5)	1stExon	Island	-2.024	0.015
cg26391832	chr2	SIX2	TSS1500	S_Shore	2.042	0.028
cg21672292	chr8	PLEC1	Body	Island	2.097	0.039
cg26590664	chr19	ZNF833	TSS200	N_Shore	2.114	0.030
cg21771200	chr19	ZNF833	TSS200	N_Shore	2.270	0.028
cg02147681	chr17	RAI1	5'UTR	Island	2.235	0.035
cg03654304	chr16	ZNF598	Body	Island	2.287	0.038
cg18886444	chr3	USP4	TSS1500	S_Shore	2.366	0.038
cg26353296	chr3	USP4	TSS1500	S_Shore	2.381	0.034
cg06406458	chr10	DOCK1	Body	OpenSea	2.028	0.044
cg26987690	chr7	UNC84A	Body	S_Shore	2.037	0.037
cg08287334	chr19	KLF16	Body	Island	2.230	0.049
cg19586199	chr19	PRKACA	TSS200; body	N_Shelf	2.582	0.030

Chr: Chromosome.

### Systematic review of published findings

#### Study selection

**Figure 1:**
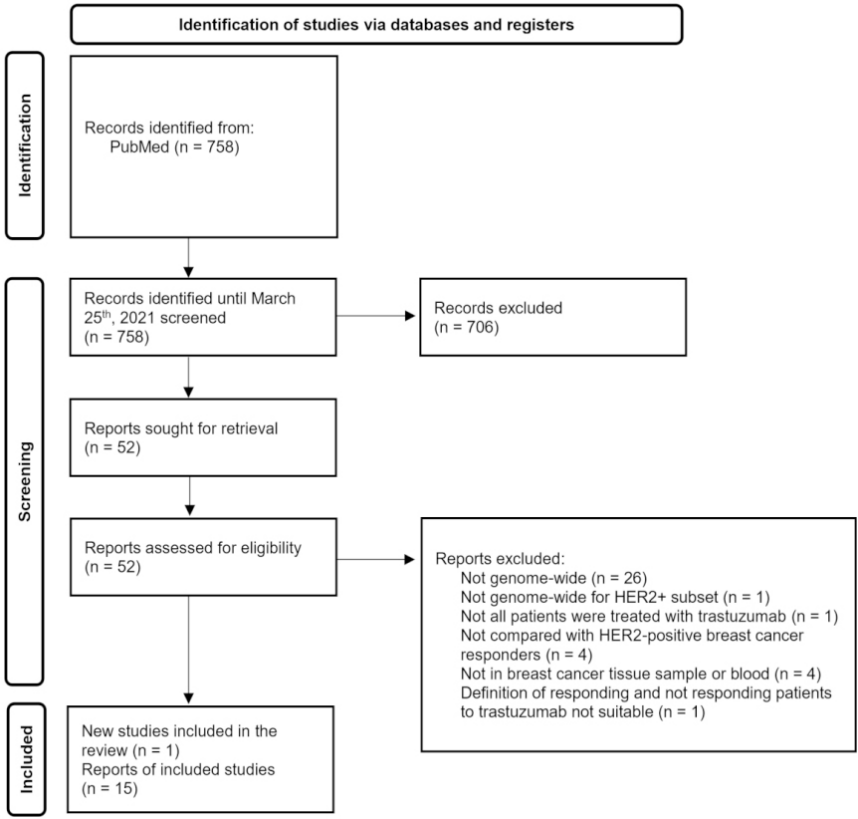
Process flow for article selection (PRISMA 2020 flow diagram).

Of the 758 references retrieved by electronic search in PubMed, we reviewed 52 full-text documents, and fifteen met the eligibility criteria [Figure 1]. This review also included our pilot study that assessed the association between DNA methylation patterns in breast cancer specimens and response to trastuzumab in a cohort of 12 HER2-positive breast cancer patients.

#### Study characteristics

In all selected studies, epigenetic and genetic patterns were measured genome-wide in breast cancer tissues or blood. DNA methylation patterns were evaluated in one study (our pilot study), gene expression profile in nine studies^[45-53]^, miRNA expression in two studies^[54,55]^, long intergenic non-coding RNA (lincRNA) profile in one study^[50]^, copy number alteration (CNA) profile in two studies^[48,56]^, protein expression in one study^[57]^ and mutations in two studies^[58,59]^. One study reported both genome-wide gene expression and genome-wide CNA patterns^[48]^, and one reported both genome-wide gene and genome-wide lincRNA expression profiles^[50]^. The majority of studies were retrospective (*n *= 10)^[46,47,49-51,54-57]^ including ours, and six studies were prospective^[45,48,52,53,58,59] ^[Table 3].

**Table 3:** Summary characteristics of studies reporting on the association between genome-wide epigenetic and genetic modifications and response to HER2-targeted therapies in HER2-positive breast cancer patients (*n *= 16)

**Study design**	**Prospective studies: ** *n *= 6 (Harris^[45]^, Guarneri^[48]^, Shi^[59]^, Sorokin^[52]^, Perez^[53]^, Lesurf^[58]^) **Retrospective studies: ***n *= 10 (Khoury^[46]^, G*á*mez-Pozo^[47]^, Triulzi^[49]^, Du^[54]^, Merry^[50]^, Ohzawa^[55]^, Zhao^[51]^, Yang^[57]^, Walsh^[56]^, Furrer [2022])
**HER2-positive breast cancer patients**	**Number of patients: **9 to 849 **Age:** median - 50 to 59 y (*n *= 6) (Gámez-Pozo^[47]^, Triulzi^[49]^, Ohzawa^[55]^, Walsh^[56]^, Yang^[57]^, Lesurf^[58]^); mean - 51.3 to 55.2 y (*n *= 2) (Furrer [2022], Sorokin^[52]^); < 45 y in 61% (*n *= 1) (Harris^[45]^); < 50 y in 48.3% (*n *= 1) (Perez^[53]^); < 65 y in 90.3% (*n *= 1) (Shi^[59]^); NR (*n *= 5) (Khoury^[46]^, Merry^[50]^, Guarneri^[48]^, Zhao^[51]^, Du^[54]^) **Stage: ** Non metastatic I-III: *n *= 1 (Du^[54]^) Non metastatic II-III: *n *= 4 (Harris^[45]^, Ohzawa^[55]^, Sorokin^[52]^, Furrer [2022]) Non metastatic NR: *n *= 3 (Guarneri^[48]^, Triulzi^[49]^, Perez^[53]^) Metastatic IV: *n *= 2 (Gámez-Pozo^[47]^, Walsh^[56]^) Non metastatic and metastatic: *n *= 1 (Khoury^[46]^) NR: *n *= 5 (Merry^[50]^, Shi^[59]^, Zhao^[51]^, Yang^[57]^, Lesurf^[58]^) **Treatment received:** Neoadjuvant NVB and T: *n *= 1 (Harris^[45]^) Adjuvant T: *n *= 1 (Khoury^[46]^) Adjuvant or neoadjuvant CT (ATC-based, TAX-based, ATC + TAX, Other CT) and T: *n *= 1 (Gámez-Pozo^[47]^) Neoadjuvant CT (PTX and FEC) plus either T (arm A), L (arm B), or T + L (arm C): *n *= 1 (Guarneri^[48]^) Neoadjuvant PTX plus either T, L, or T + L: *n *= 1 (Shi^[59]^) Adjuvant CT and T: *n *= 4 (Triulzi^[49]^, Du^[54]^, Merry^[50]^, Ohzawa^[55]^) Neoadjuvant CT and T: *n *= 2 (Zhao^[51]^, Lesurf^[58]^) Adjuvant T only or T plus DTX/PTX + CBDCA/PTX/DTX + CBDCA/Cap/NVB/Gem: *n *= 1 (Sorokin^[52]^) Neoadjuvant therapy or surgical treatment and T: *n *= 1 (Yang^[57]^) T: *n *= 1 (Walsh^[56]^) Adjuvant ATC + CTX, PTX, T, or ATC + CTX, PTX + T, T: *n *= 1 (Perez^[53]^)
**Genome-wide profiling method**	**Methylation: ** *n *= 1 (Furrer [2022]) **miRNA expression: ***n *= 2 (Du^[54]^, Ohzawa^[55]^) **Gene expression: ***n *= 9 (Harris^[45]^, Khoury^[46]^, Gámez-Pozo^[47]^, Guarneri^[48]^, Triulzi^[49]^, Merry^[50]^, Zhao^[51]^, Sorokin^[52]^, Perez^[53]^) **CNA: ***n *= 2 (Guarneri^[48]^, Walsh^[56]^) **lincRNA expression: ***n *= 1 (Merry^[50]^) **Protein expression: ***n *= 1 (Yang^[57]^) **Mutations: ***n *= 2 (Lesurf^[58]^, Shi^[59]^)
**Outcome**	**Disease-free survival (DFS): ** *n *= 4 (Khoury^[46]^, Du^[54]^, Sorokin^[52]^, Furrer [2022]) **Relapse-free survival (RFS): ***n *= 2 (Perez^[53]^, Triulzi^[49]^) **Pathological complete response (pCR): ***n *= 10 (Harris^[45]^, Gámez-Pozo^[47]^, Guarneri^[48]^, Merry^[50]^, Shi^[59]^, Ohzawa^[55]^, Zhao^[51]^, Yang^[57]^, Walsh^[56]^, Lesurf^[58]^)

ATC: Anthracycline; Cap: capecitabine; CBDCA: carboplatin; CNA: copy number alteration; CT: chemotherapy; CTX: cyclophosphamide; DTX: docetaxel; FEC: fluorouracil, epirubicin, and cyclophosphamide; Gem: gemcitabine; L: lapatinib; NVB: vinorelbine; NR: not reported; PTX: paclitaxel; T: trastuzumab; TAX: taxane; y: years.

DFS was reported in four studies including ours^[46,52,54]^, RFS in two studies^[49,53]^, and pCR in ten studies^[45,47,48,50,51,55-59]^. DFS was defined as the time between the diagnosis and recurrence (locoregional recurrence, recurrence in the contralateral breast, and distant breast cancer recurrence) or death; RFS was defined as the time from the start of trastuzumab treatment to the first local, regional or distant recurrence event; and pCR as the absence of invasive breast cancer in the breast and axillary lymph nodes at the time of surgery. Besides ours, the other fifteen included studies were published between 2007 and 2020 and involved between 9 and 849 HER2-positive, anti-HER2 therapy-treated breast cancer patients [Table 3].

#### Studies of genome-wide DNA methylation and response to targeted treatment

Characteristics of the study (our new findings) that evaluated the association between genome-wide DNA methylation patterns measured in breast cancer tissues and response to targeted treatment in HER2-positive, trastuzumab-treated breast cancer patients are presented in Supplementary Table 3. The study was retrospective. The mean age of included HER2-positive breast cancer patients was 51.3 years. The proportion of ER-positive breast cancer patients was 67%, and the proportion of positive lymph node status was 92%. Sixty-seven percent of HER2-positive breast cancer patients were postmenopausal. DNA methylation was assessed using Infinium HumanMethylation450 BeadChip array. Breast cancer patients were treated with adjuvant chemotherapy and trastuzumab. The study reported DFS. HER2-positive breast cancer patients were of Caucasian ethnicity. Tumor cell fraction was ≥ 70%.

#### Studies of genome-wide gene expression profile and response to targeted treatment

Characteristics of the nine studies that examined the association between genome-wide gene expression profiles measured in breast cancer tissues and response to HER2-targeted therapies in HER2-positive breast cancer patients are presented in Supplementary Table 4. Five studies were retrospective^[46,47,49-51]^, and four studies were prospective^[45,48,52,53]^. The median age of HER2-positive breast cancer patients was not reported in four studies^[46,48,50,51]^ and varied from 52 to 58 years in two studies^[47,49]^. The mean age was 55.2 years in one study^[52]^, 60.9% of participants were < 45 years in another^[45]^, and 48.3% of participants were < 50 years in one other study^[53]^. The proportion of ER-positive breast cancer patients was reported in three studies and varied between 43.4% and 69.6%^[45,47,49]^. In eight studies, patients received trastuzumab^[45-47,49-53]^, and in the remaining study, patients were treated with trastuzumab, lapatinib or both^[48]^. pCR was reported in five studies^[45,47,48,50,51]^, RFS in two studies^[49,53]^, and DFS in two studies^[46,52]^. Gene expression profile was evaluated in fresh frozen tissue in five studies^[45,46,48,50,51]^ and in FFPE tissues in four studies^[47,49,52,53]^. Gene expression profile was assessed using HumanHT-12 v3 BeadChip^[46]^, HumanHT-12 v4 BeadChip^[49]^, Affymetrix Human Genome U219 array^[47]^, Affymetrix GeneChip 3’ IVT Express kit^[48]^, Illumina HiSeq 2500 platform^[50]^, Illumina HiSeq 3000^[52]^, GeneChip U133 Plus 2.0 Gene Array^[45]^, DASL technology^[53]^ and Agilent UNC Perou Lab Homo sapiens 1X44K Custom Array/Illumina HumanHT-12 WG-DASL V4.0 R2 expression BeadChip/Affymetrix technology using the HG-U133 Plus 2.0 GeneChip array^[51]^. Tumor cell fraction was not reported in five studies^[47,48,50,51,53]^, and was at least 70% in three studies^[46,49,52]^. In the remaining study, breast cancer samples of each patient contained at least 500 breast cancer cells^[45] ^[Supplementary Table 4]. 

#### Study of genome-wide miRNA expression profile and response to targeted treatment

Characteristics of the two studies that examined the association between genome-wide miRNA expression profile and response to targeted treatment in HER2-positive trastuzumab-treated breast cancer patients are presented in Supplementary Table 5. Both studies had a retrospective design^[54,55]^. miRNA expression was measured genome-wide in FFPE breast cancer tissues using Agilent miRNA assay^[54]^ or Agilent SurePrint G3 Human miRNA microarray^[55]^ in cohorts of 14 and 40 HER2-positive breast cancer patients. Patients were treated with trastuzumab. DFS^[54]^ and pCR^[55]^ were reported. Tumor cell fraction was not reported in one study^[54]^ and was at least 80% in the other^[55]^.

#### Study of genome-wide lincRNA expression profile and response to targeted treatment

Characteristics of the single study that examined the association between genome-wide lincRNA expression profile and response to targeted treatment in HER2-positive trastuzumab-treated breast cancer patients are presented in Supplementary Table 6. The study had a retrospective design. lincRNA expression was measured genome-wide in fresh frozen breast cancer tissues using the Illumina HiSeq 2500 platform in a cohort of 13 HER2-positive breast cancer patients. Patients were treated with trastuzumab and pCR was reported. Tumor cell fraction was not reported.

#### Study of genome-wide CNA profile and response to targeted treatment

Characteristics of the two studies that examined the association between genome-wide CNA profile and response to HER2-targeted therapies in HER2-positive breast cancer patients are presented in Supplementary Table 7. The studies had a prospective design^[48,56]^. CNA profile was assessed genome-wide in fresh frozen breast cancer tissues using Affymetrix Genome-wide Human SNP array^[48]^ or Illumina HiSeq^[56]^ in cohorts of 68 and 11 HER2-positive breast cancer patients. Patients were treated with trastuzumab, lapatinib or both in one study^[48]^ and trastuzumab in the other^[56]^. pCR was reported. Tumor cell fraction was not reported.

#### Study of genome-wide protein expression profile and response to targeted treatment

Characteristics of the only study that examined the association between genome-wide protein expression profile and response to targeted treatment in HER2-positive trastuzumab-treated breast cancer patients are presented in Supplementary Table 8. The study had a retrospective design^[57]^. The protein expression profile was assessed genome-widely in blood using TMT-6 plex Isobaric Label Reagent Set in a cohort of 6 HER2-positive breast cancer patients. Patients were treated with trastuzumab. pCR was reported. 

#### Study of genome-wide mutation profile and response to targeted treatment

Characteristics of the two studies that examined the association between genome-wide somatic or germline mutation profile and response to targeted treatment in HER2-positive breast cancer patients are presented in Supplementary Table 9. The studies had a prospective design^[58,59]^. Somatic mutation profile was assessed genome-wide in fresh frozen breast cancer tissues for the two studies. Germline mutation profile was assessed genome-widely in blood using Illumina HiSeq 2000^[59]^, and whole genome sequencing (WGS) and whole exome sequencing (WES) + Illumina HiSeq 2000 platform^[58]^ in cohorts of 48 and 203 HER2-positive breast cancer patients. Patients were treated with trastuzumab. pCR was reported. Tumor cell fraction was not reported in one study^[58]^ and was at least 10% in the other study^[59]^.

#### Risk of bias in studies

Overall, studies ranged on average from low to critical risk of bias [low (*n *= 2)^[53,59]^, moderate (*n *= 4, including our study)^[47,49,57]^, serious (*n *= 1)^[54]^, and critical (*n *= 9)]^[45,46,48,50-52,55,56,58]^, most commonly due to confounding. The bias evaluation of each included study is presented in Supplementary Table 10. 

### Results of individual studies

#### Genome-wide gene expression profile in breast cancer tissues and response to anti-HER2 agents in HER2-positive breast cancer patients

The genes reported to be differentially expressed in breast cancer tissues of cases compared to controls for each identified study are presented in Supplementary Table 11. Higher expression of the following genes was consistently observed in two different studies: *ESR1*^[49,50]^, *RBP1*, *SLP1*^[46,50]^, *EPS8L1*^[50,52]^, *MEP1B*, *UTP15*, *RRAS2*, *GRB10*, *FAM98A*, *RBMS2*, *RPL9*, *RPAIN*, *MORF4L1*, *LLPH*, *MTMR1*, *FRYL*, *FLCN*, *CLINT1*, *ERICH1*^[47,52]^, *ZNF281*, *ANKRD52*, *PIAS3*, *BRD1*, *RNF146*, *CLDN12*, *PROM2*, *COMMD5*, *VMP1*, *DEDD*, *ZNF439*, *CRIP2*, *PRSS16*, *GUK1*, *RRN3*, *PLEKHG3*, *JUN*, *BCL9*, *SLC25A37*, *CRYZ*, *RNF24*, *PSMG3*, *PAQR7*, *ABT1*, *WNT7B*, *SLC35B2*, *SYTL4*, *NUPR1*, *DPY19L1*, *DAZAP1*, *EEF1D*, *SGPP1*, *GALNT2*, *SPA17*, *RAD51*, *MBD6*, *KIF1C*, *C1QTNF3*, *BLOC1S2*, *SLC2A10*, *ZNF740*, *ADCK2*, *SLC41A1*, *RAB4A*, *CRIP1*, *ZNF552*, *CARHSP1*, *POFUT1*, *EMC10*, *BAX*, *HOXC4*, *DDR1*, *CTSD*, *FEN1*, *SULT1A1*, *DUSP14*, *IRF9*, *TMC4*, *MUC1*, *LMAN2*, *LASP1*, *SHROOM1*^[50,52]^, *NSL1*, *ENAH*, *UBE2Q2*, *GNPAT*, *THBS2*, *TBCEL*, *FAM46A*, *ZNF678*, *TSEN15*, *ZNF674*, *CNIH4*, *ASAH1*, *SELT*, *ARFGAP3*, *TATDN3*, *FBLN1*, *MOSPD1*, *PPCS*, *NUCKS1*, *PGBD2*, *ACBD3*, *ORMDL2*, *AMMECR1*, *TNFRSF19*, *FOSL2*, *PYCR2*, *WSB1*, *TROVE2*, *RWDD3*^[47,50]^, *LYSMD3*^[47,50]^, *APOB*, *SLC3A2*, *CST3*^[52,57]^, *BOC*^[45,52]^, *DDX27*, *IL17RC*, *PKP3*, *WNK2*^[52,53] ^*AGRN*, *ATRN*, *NACC1*, *PCSK6*, *PIR*, *PLAUR*, *QSOX1*, *RHBDL2*, *SEC22B*, *SERPINH1*, *TCEAL1*^[50,53]^, *CSDE1*, *SCUBE3*, *TMEM167A*^[47,53]^*.* Lower expression of the following genes was consistently reported in two different studies: *ORMDL3*^[49,50]^, *NME1*^[46,50]^, *SLC35B1*^[46,50]^, *UTP18*^[46,50]^, *PHB*^[46,50]^, *S100B*, *TOM1L1*^[50,52]^, *HIST1H2BG*, *PRR13*^[47,50]^, *NXPH1*, *CKS2*, *NETO2*, *SLC12A2*^[46,52]^, *ACSL5*, *CD3E*, *GIMAP7*, *GZMK*, *PPARG*, *SELL*, *VCAM1*^[50,53]^, *CLEC10A*, *CTLA4*, *FGL2*, *TSPAN7*^[52,53]^*, DENND3*, *TIAM1*^[47,53]^. The following genes were reported to be higher expressed in breast cancer samples of cases compared to controls in two different studies but were reported to be lower expressed in another study: *SFRP1*^[45,46,50]^, *ACTR1B*, *FASTK*, *TMEM219*, *NDUFA3*^[47,50,52]^, *ATP5I*^[47,50,52]^. The following genes were reported to be lower expressed in breast cancer samples of cases compared to controls in two different studies but were reported to be higher expressed in another study: *PABPC1*^[47,50,52]^, A*RPC1A*, *TXN*^[47,50,52]^, *TOB1*^[46,50,52]^. Higher expression of the *GOLGA2*^[47,50,52]^ and *PHF21A*^[47,50,52]^ genes was consistently observed in three different studies. 

Gámez-Pozo *et al.* and Sorokin *et al.* identified several pathways associated with response to trastuzumab, including those involved in EGF receptor signaling, PI3K, apoptosis signaling, and p53^[47,52]^. Gámez-Pozo *et al.* observed that the PI3K pathway was the most strongly associated with treatment response^[47] ^[Supplementary Table 12]. Sorokin *et al.* identified several pathways associated with response to trastuzumab. The most statistically and significantly upregulated ones in the trastuzumab-sensitive group were PPAR Pathway and cAMP Protein Retention Pathway^[52]^. Triulzi *et al.* and Sorokin *et al.* reported that breast cancer samples of patients with a lower risk of early relapse showed higher expression of genes enriched in immune system-related pathways and proliferation-associated pathways^[49,52]^. For two^[46,48]^ out of the four included studies that did not report pathway analysis^[45,46,48,50]^, we performed gene ontology analysis using the list of genes reported as being differentially expressed by the authors using PANTHER. We observed that the Notch signaling pathway was overrepresented in both studies^[46,48]^, an observation also reported by Sorokin *et al.*^[52]^. We observed that Wnt signaling was overexpressed in Khoury *et al.*^[46]^, as reported by Sorokin *et al.*^[52]^. We did not perform pathway analysis for the two remaining studies^[45,53]^, as the number of differentially expressed genes (*n *= 11) was too small to perform the analysis^[45]^ or not reported^[53]^.

In our study, we observed overlap between the identified differentially expressed genes and the strongly differentially methylated (i.e., |log2FC| > 2.0) genes. *PRKACA* was hypermethylated in our study (within the TSS region as well as the gene body), upregulated in one study^[50]^, and downregulated in another study^[47]^.

#### Genome-wide miRNA expression profile in breast cancer tissues and response to trastuzumab in HER2-positive breast cancer patients treated with trastuzumab

Du *et al.* identified seven upregulated and two downregulated miRNAs in breast cancer tissues of cases compared to controls^[54]^. Ohzawa *et al.* identified four upregulated and ten downregulated miRNAs in breast cancer tissues of cases compared to controls^[55] ^[Supplementary Table 13]. Regarding the miRNAs identified by Du *et al.*, 902 genes were predicted to be targeted by miR-150-5p, 47 genes by miR-4734, 570 genes by miR-361-5p, 1,134 genes by miR-26a-5p, 416 genes by miR-365a-3p, 701 genes by miR-155-5p, 737 genes by miR-205-5p, 1,384 genes by miR-106b-5p, and 187 genes by miR-424-3p (as illustrated in a database for miRNA target prediction and functional annotations available online at www.mirdb.org)^[54]^. For the miRNAs reported by Ohzawa *et al.*, 484 genes were predicted to be targeted by miR-210, 242 genes by miR-31-3p, 891 genes by miR-449a, 801 genes by miR-449b-5p, 21 genes by miR-106b-3p, 263 genes by miR-1180, 242 genes by miR-1238-5p, 1,133 genes by miR-142-5p, 902 genes by miR-150-5p, 1,409 genes by miR-181c-5p, 1,266 genes by miR-182-5p, 438 genes by miR-20a-5p, 1,084 genes by miR-218-5p, 1,249 genes by miR-3609, 270 genes by miR-362-5p, 420 genes by miR-3620-3p, 676 genes by miR-4418, 272 genes by miR-4506, 410 genes by miR-4657, 406 genes by miR-505-3p, and 392 genes by miR-505-5p^[55]^. 

We observed that among the 344 genes that were reported to be differentially expressed (at the mRNA level) between breast cancer tissues of cases and controls in at least two studies, 170 were predicted to be targeted by miRNA identified as differentially expressed between breast cancer tissues of cases and controls in Ohzawa *et al.*^[55]^ or Du *et al.*^[54] ^[Supplementary Table 14]. We observed that among the 15 genes identified as differentially methylated in our study, five (*KCNH7*, *ADAMTS2*, *SIX2*, *DOCK1* and *ZNF598*) were predicted to be targeted by miRNA identified as differentially expressed in breast cancer tissues of cases compared to controls in the study of Ohzawa *et al.*^[55]^ or Du *et al.*^[54] ^[Supplementary Table 14]. 

#### Genome-wide lincRNA expression profile in breast cancer tissues and response to targeted treatment in HER2-positive breast cancer patients treated with trastuzumab

Merry and collaborators observed that 371 lincRNAs were differentially expressed in non-pCR samples compared to pCR samples, where 33 lincRNAs showed decreased expression and 338 increased expression^[50] ^[Supplementary Table 15]. Among these 371 genes, 97 were reported to be differentially expressed at the mRNA level in breast cancer tissues of cases compared to controls in at least two studies: *FAM84B*, *PHF21A*, *NDUFV3*, *COMMD6*, *SRP9*, *S100B*, *WDR26*, *LYSMD3*, *CSTB*, *C5orf39*, *FOXA1*, *GALM*, *ITGB2*, *SP140*, *UTRN*, *SAA2*, *SHB*, *ZFP64*, *ZC3H12B*, *NADSYN1*, *B4GALNT4*, *AP2B1*, *USP16*, *ARL4D*, *SYNPO2*, *FAIM3*, *CBR1*, *EFR3B*, *IL18*, *LOC389493*, *FBXO16*, *FAM114A1*, *MAP3K9*, *TOX3*, *ZNF681*, *IDH3B*, *TPBG*, *PRKACB*, *UBE2A*, *ID2*, *IRX3*, *CILP*, *COL5A2*, *WRB*, *MBOAT1*, *GCA*, *SATB2*, *HERC6*, *RALGPS2*, *NUF2*, *MIA3*, *FAM91A1*, *FAM5C*, *C1orf227*, *RPP4*, *MYOT*, *PRKAA2*, *MAP1LC3B*, *PEX3*, *MEST*, *F13A1*, *CREB1*, *LRCH2*, *PCF11*, *POLR3G*, *RORA*, USP3, TSHZ3, CXCR4, *CCNH*, *CCM2*, *ZNF814*, *RAPH1*, *ZPBP2*, *FBXW4*, *ODF3B*, *CROCC*, SH3RF2, *HEATR6*, *CDK13*, *ATF3-1*, *ATF3*, *DTL*, *IARS2*, RC3H1, RC3H1, *URB2*, *RHOU*, *RC3H1*, *RC3H1*, *SUPT3H*, *ABI1*, *OTUD7B*, *GPATCH2*, *RNF2*, *IRF2BP2*.

Among the differentially expressed lincRNAs reported by Merry *et al.*^[50]^, we observed 44 genes that were predicted to be target genes of miRNA identified as differentially expressed in the study of Ohzawa *et al.*^[55]^ or Du *et al.*^[54] ^[Supplementary Table 14]. 

None of the genes whose lincRNAs were reported to be differentially expressed between patients showing pCR and those showing non-pCR in Merry *et al.* was reported as differentially methylated with a |log2FC| > 2.0 between cases and controls in our study^[50]^. However, when we consider the entire list of differentially methylated genes in our study, regardless of log_2_-fold change, we identified six overlapping genes. Among these six genes, five were hypermethylated (*GABRA5*, *ZIC5*,* GRAMD4*,* RSPH3*, and* VCAN*), and one was hypomethylated (*CSMD1*) in breast cancer samples of cases compared to controls. 

#### Genome-wide copy number alterations in breast cancer tissue and response to targeted treatment in HER2-positive breast cancer patients treated with anti-HER2 agents

In their analysis of the association of genome-wide CNA in breast cancer tissues of HER2-positive breast cancer patients who received trastuzumab, lapatinib, or both, with response to anti-HER2 treatment, Guarneri *et al.* reported that, unlike pCR patients, non-pCR patients showed a CNA signature^[48]^. Overall, the authors observed CN alterations, mainly CN gains, in 557 genes located on chromosomes 1, 8, 17, 20 [Supplementary Table 16]. We observed that among the 344 genes that were reported to be differentially expressed in breast cancer tissues of cases compared to controls in at least two studies, 23 genes showed CN alterations in non-pCR samples compared to pCR-samples in Guarneri *et al.*^[48]^: *FAM84B*^[46,48]^, *SRP9*^[50,52]^, *WDR26*^[47,50]^, *BATF3*^[47,50]^, *EDARADD*, *ENPP2*, *LAX1*, *NUAK2*, *PTPRC*^[53]^, *KIF21B*, *RNF19A*, *ZNF831*, *NR5A2*^[52,53]^, *KIF26B*, *LAMB3*, *C1orf133*^[50,53]^, *LAMC2*^[52,53]^, *FAIM3*^[50]^, *MIA3*^[47]^, *OTUD7B*, *GPATCH2*, *RNF2*, *IRF2BP2*^[50]^.

We observed that among the 557 genes that were reported to show CN alteration in non-pCR samples compared to pCR-samples in the study of Guarneri *et al.*^[48]^, 279 were predicted to be targeted by miRNA identified as differentially expressed in breast cancer tissues of cases compared to controls in the study of Du *et al.*^[54]^ or Ohzawa *et al.*^[55] ^[Supplementary Table 14]. 

Among the genes showing CN alterations in non-pCR samples compared to pCR-samples in the study of Guarneri *et al.*^[48]^, 18 genes (*FAM84B*, *SRP9*, *WDR26*, *MIA3*, *FAM91A1*, *FAM5C*, *C1orf227*, *ATF3*, *DTL*, *IARS2*, *RC3H1*, *URB2*, *RHOU*, *RC3H1*, *OTUD7B*, *GPATCH2*, *RNF2*, *IRF2BP2*) showed higher lincRNA expression and one (*FAIM3)* showed lower lincRNA expression in Merry *et al.*^[50]^. 

No overlap was observed between genes showing CN alterations in non-pCR samples compared to pCR-samples in Guarneri *et al.* and genes identified as differentially methylated with a |log2FC| > 2.0 in our study^[48]^. However, when we consider the whole list of differentially methylated genes, we identified 27 genes that were differentially methylated in breast cancer tissues of cases compared to controls in our study among the 557 genes showing CNV variations in the study of Guarneri *et al.*^[48]^. Of these 27 differentially methylated genes, nine were hypomethylated (*PLD5*, *GJD2*, *C20orf85*, *APCDD1L*,* VASH2*, *PSEN2*,* FMN2*,* EDN3*,* ACTN2*) and 18 were hypermethylated (*TRAF5*, *TSEN15*, *TRIB1*, *TMEM206*, *SNX31*, *SMG7*, *RGS1*, *PRG4*, *PGBD5*, *NID1*, *KCNV1*, *GPATCH2*, *EXT1*, *CDK18*, *CAPN9*, *ADSS*, *ABR*, *C1orf55*). 

#### Genome-wide protein expression profile in blood of breast cancer cases compared to controls

Out of the 18 genes that were reported as differentially expressed (five downregulated and 13 upregulated) in the blood of breast cancer cases compared to controls by Yang *et al.*^[57] ^[Supplementary Table 17], three (*APOB*, *SLC3A2*, *CST3*) were differentially expressed in breast cancer tissues of cases compared to control in at least two studies. Three (*LDHA*, *DBF4B*, and *MASP1*) were predicted to be targeted by miRNA identified as differentially expressed in breast cancer tissues of cases compared to controls in the study of Du and collaborators^[54]^ or Ohzawa and collaborators^[55] ^[Supplementary Table 14]. 

None of the differentially expressed genes in the study of Yang *et al.* were differentially methylated in our study^[57]^.

#### Genome-wide somatic and germline mutations profile in breast cancer tissues of cases compared to controls

Whereas Shi and collaborators observed that higher somatic mutation frequency in the *PIK3CA* gene in the breast tissues of cases was associated with trastuzumab resistance^[59]^, Lesurf *et al.* reported that no somatic or germline mutations were associated with response to trastuzumab in breast cancer tissues of cases compared to controls^[58] ^[Supplementary Table 18].

## DISCUSSION

In a cohort of 12 HER2-positive breast cancer patients treated with trastuzumab, interrogation of DNA methylation using the Infinium HumanMethylation450 BeadChip allowed identifying genes that were differentially methylated between trastuzumab-resistant and trastuzumab-sensitive HER2-positive breast cancer patients. Interestingly, among the strongly differentially methylated genes, we observed genes associated with human cancer, including *DOCK1*, *ADAMTS2*, *PLEC1*, *USP4*, and *PRKACA*^[60-69]^. 

The guanine nucleotide exchange factor *DOCK1* (Dedicator of cytokinesis protein 1) is involved in cytoskeletal rearrangements required for phagocytosis of apoptotic cells and cell mobility^[70]^. A recent study reported that *DOCK1* inhibition leads to suppressed migration of the triple-negative breast cancer cell lines MDA-MB-157 and MDA-MB-231^[68,71]^. *ADMATS2* (ADAM metallopeptidase with thrombospondin type 1 motif 2) belongs to the ADAM metallopeptidase with thrombospondin type 1 motif and processes collagen precursors into mature collagen molecules^[72]^. It has been proposed that *ADAMTS2* exerts an anti-tumor effect by inhibiting intratumoral vascularization^[73]^. The *PLEC* gene encodes the pectin protein, which plays a role in maintaining tissue integrity^[74]^. A recent study suggests that a *PLEC* gene polymorphism (rs138924815) might increase the risk for familial testicular cancer^[75]^. *USP4* (Ubiquitin carboxyl-terminal hydrolase 4) is a deubiquitinating enzyme that removes conjugated ubiquitin from target proteins^[76]^. It has been reported that *USP4* expression was decreased in breast cancer tissue samples compared to paired normal breast tissues^[63]^. Moreover, *USP4* expression was associated with decreased proliferation in two HER2-negative breast cancer cell lines (MCF7 and BT549)^[63]^. The *PRKACA* gene encodes for a protein kinase that plays a role in controlling cellular processes such as glucose metabolism and cellular division^[72]^. Of note, one recent study suggests that *PRKACA* expression might be associated with the development of trastuzumab resistance in HER2-positive breast cancer patients^[64]^. The authors observed that in a subgroup of HER2-positive breast cancer patients who developed trastuzumab resistance (three out of five patients), *PRKACA* expression was highly increased in the breast cancer sample obtained after the onset of trastuzumab resistance compared to the pre-treatment sample. Considering that in our study, the *PRKACA* gene was hypermethylated within the gene body and that hypermethylation within this gene region often promotes gene elongation and, therefore, gene expression, we can postulate that our results might be concordant with those reported by Moody *et al.*^[64]^. Although in our study, DNA methylation was exclusively measured in pre-treatment samples and Moody *et al.* observed increased *PRKACA* expression only in breast cancer samples obtained after the onset of recurrence^[64]^. 

Among all genes that we identified as strongly differentially methylated between trastuzumab-resistant and trastuzumab-sensitive HER2-positive breast cancer patients, one of them, *PRKACA*, was reported as being higher expressed in breast cancer tissues of cases compared to controls in the study conducted by Merry and collaborators^[50]^ and as being lower expressed in breast cancer tissues of cases compared to controls in the study of Gámez-Pozo *et al.*^[47]^. In our study, the *PRKACA* gene was hypermethylated within the TSS region and the gene body. Our observation could be partly concordant with the results reported by Merry *et al.*^[50]^ (but not with those of Gámez-Pozo *et al.*^[47]^), as hypermethylation in the gene body (but not within the TSS region) is usually associated with increased gene expression^[34]^. Interestingly, the results of Merry and collaborators^[50]^ (but not those of Gámez-Pozo *et al.*^[47]^) are concordant with the study above^[64]^, where the authors observed that *PRKACA* expression was increased in breast cancer samples of HER2-positive trastuzumab-resistant breast cancer patients.

In one of the studies retained in our systematic review, the authors created a predictive model to differentiate HER2-positive trastuzumab-treated breast cancer patients with a higher risk of relapse from those with a lower risk in a cohort of 53 patients^[49]^. The validity of this predictive model was then confirmed in an independent and bigger data set. The authors observed that differentially expressed genes in the breast cancer tissues of patients identified as at low risk for relapse in the independent data set using this model were associated with the immune system. When we performed gene enrichment analysis of genes showing CN alteration in the study of Guarneri and collaborators, we observed that pathways associated with the immune response (inflammation mediated by chemokine and cytokine signaling pathway) were overrepresented. The immune system's involvement in response to anti-HER2 agents in HER2-positive breast cancer patients has also been reported in other studies^[77-82]^.

To our knowledge, we conducted the first systematic review on the association of epigenetic and genetic alterations in breast cancer tissues or blood with the response to anti-HER2 agents in HER2-positive breast cancer patients. Sixteen studies were included in this review, and very few overlaps between studies were found. The most consistent results were the higher expression of *GOLGA2* and *PHF21A* genes and the higher expression and CN gain of *MIA3*, *WDR26* and *C1orf133* genes observed in three different studies. Among these five genes, WDR26 has been shown to promote breast cancer growth and metastasis via the PI3K/AKT signaling pathway^[83]^. Gámez-Pozo and collaborators also observed that genes identified as differentially expressed in breast tumor tissues of cases compared to controls were overrepresented in the PI3K pathway^[47]^. Interestingly, the study by Shi and collaborators^[59]^ also reported that PI3K mutations were associated with response to anti-HER2 agents in HER2-positive breast cancer patients and other previously published studies^[84-86]^. Taken together, these results suggest that genes of the PI3K pathway might play a relevant role in the development of resistance to anti-HER2 agents in breast cancer patients. Exploring the ramifications of these and other findings in larger cohorts or datasets like TCGA should be considered in future studies.

Several factors might explain why we only observed a few overlaps in our systematic review. Tumor cell content varied from > 40% to > 80% between studies, and in several publications (*n *= 4), this information was not provided. Contamination with cell types other than breast cancer cells can modify the observed pattern of genetic and epigenetic markers, as DNA methylation and other epigenetic or genetic markers widely vary across tissues and cellular types^[87]^. Moreover, the type of outcome evaluated in the study might also play a role, as the assessment of pCR in the neoadjuvant setting might mainly identify patients who did not primarily respond to targeted treatment (primary resistance), whereas the evaluation of DFS in the adjuvant setting might allow identifying patients who initially respond to targeted treatment but who develop resistance over time (acquired resistance). Moreover, epigenetic and genetic markers can be influenced by clinicopathological data, including age, stage, ER status, menopausal status, and ethnicity^[88-96]^. Unfortunately, it was difficult to evaluate this aspect, as patients’ clinicopathological data were not extensively reported in the majority of the included studies. The risk of bias in most studies was due to confounding. 

## CONCLUSION

In conclusion, although the sample size of the present pilot study was small and despite the lack of validation cohort, using a high-throughput analysis, we identified genes that were differentially methylated in breast cancer tissues of HER2-positive trastuzumab-treated breast cancer patients who developed resistance toward this drug compared to those who responded to targeted therapy. One of the most differentially methylated genes, *PRKACA*, has been reported to be differentially expressed in breast cancer tissues of trastuzumab-resistant compared to trastuzumab-sensitive HER2-positive breast cancer patients in two studies included in our systematic review. Although we identified very few genes that overlap between studies, our review suggests that some of the genes acting in the PI3K pathway, such as *PRKACA*^[97]^, might play an important role in developing resistance to anti-HER2 agents in breast cancer patients. Although the associations between *PI3KCA* mutations and *PI3K* dysfunctions and anti-HER2 treatment resistance are well documented in the literature^[86,98-100]^, further studies on this topic are needed, which may help to unveil carcinogenic mechanisms involved in this pathway.

Although the observations reported in the retained studies were only marginally concordant, our work and the studies presented in this article suggest that knowledge gathered from these high-throughput studies could be useful for the identification of novel biomarkers of trastuzumab resistance. This might promote the development of new targeted drugs that could be administered to trastuzumab-resistant HER2-positive breast cancer patients.

## DECLARATIONS

### Availability of data and materials

All data generated or analysed during this study are included in this published article [and its supplementary information files].

### Copyright

© The Author(s) 2022.
