# Supplementary material for: Association between genome-wide epigenetic and genetic alterations in breast cancer tissue and response to HER2-targeted therapies in HER2-positive breast cancer patients: new findings and a systematic review
Source: Cancer Drug Resist. 2022 Nov 2;5(4):995–1015. doi: 10.20517/cdr.2022.63 (PMC9771759; doi:10.20517/cdr.2022.63)
Supplement: Supplementary file 1 [file cdr-5-4-995-SupplementaryMaterials.pdf]

Supplementary Materials

Association between genome-wide epigenetic and genetic alterations in breast cancer tissue and response to HER2-targeted therapies in HER2-positive breast cancer patients: new findings and a systematic review

Supplementary Table 1. Baseline characteristics of selected cases (*n* = 6) compared to those of the total population of cases (*n* = 22)

**Factor**	**Selected cases (*n* = 6)**	**Total population of cases (*n* = 22)**	***P*-value**
	Mean ± SD	Mean ± SD	
Age (years)	51.3 ± 6.1	53.9 ± 10.9	0.87
Body mass index (kg/m^2)	23.8 ± 2.6	25.9 ± 4.4	0.94
Follow-up time (years)	3.8 ± 2.8	4.0 ± 2.9	0.87
	n (%)	*n* (%)	
Grade			
I/II	2 (33%)	12 (54%)	1.00
III	4 (67%)	9 (41%)	
Missing value	0 (0%)	1 (5%)	
Lymph node status			
Negative	0 (0%)	6 (27%)	1.00
Positive	6 (100%)	16 (73%)	
Tumor size (cm)			
≤ 5	5 (83%)	20 (91%)	1.00
> 5	1 (17%)	2 (9%)	
Estrogen receptor status			
Negative	2 (33%)	10 (45%)	1.00
Positive	4 (67%)	12 (55%)	
Progesterone receptor status			
Negative	3 (50%)	13 (59%)	1.00
Positive	3 (50%)	9 (41%)	

Menopausal status			
Pre	2 (33%)	5 (23%)	1.00
Post	4 (67%)	17 (77%)	
Radiotherapy			
No	1 (17%)	8 (36%)	1.00
Yes	5 (83%)	14 (64%)	
Endocrine Therapy			
No	2 (33%)	10 (45%)	1.00
Yes	4 (67%)	12 (55%)	
Chemotherapy			
No	0 (0%)	0 (0%)	1.00
Yes	6 (100%)	22 (100%)	
Trastuzumab treatment completed			
No	0 (0%)	4 (18%)	1.00
Yes	6 (100%)	18 (82%)	

n: Number of subjects; SD: standard deviation.

Supplementary Table 2. Search strategy in PubMed database

Database/Consultation date	#	Concept	Search strategy	Results	Combination of concepts	
					#	Total
Pubmed/ 20210325	#1	Population = breast cancer patients	(("breast neoplasms"[Mesh]) OR (("breast"[tiab] OR "mammary"[tiab]) AND ("cancer"[tiab] OR "cancers"[tiab] OR "carcinoma*"[tiab] OR "neoplasm"[tiab] OR "neoplasms"[tiab] OR "tumor*"[tiab] OR "tumour*"[tiab] OR	446,644	#1 AND #2 AND #3 AND #4 AND #5	758

			"malignancy"[tiab] OR "malignancies"[tiab])))			
	#2	Population = HER2-positive type of BC	("HER2(776-788)" [Supplementary Concept] OR "Receptor, ErbB-2"[Mesh] OR "HER2"[tiab] OR "HER2 positive"[tiab] OR "HER-2 positive"[tiab] OR "human epidermal growth factor receptor 2-positive"[tiab] OR "erbb2 positive"[tiab] OR "erbb-2 positive"[tiab])	42,156		
	#3	Population = treated with anti-HER2 agent	(Trastuzumab[Mesh] OR Lapatinib[Mesh] OR gefitinib[Mesh] OR erlotinib[Mesh] OR "Antibodies, Monoclonal, Humanized"[Mesh] OR Trastuzumab[tiab] OR Lapatinib[tiab] OR gefitinib[tiab] OR erlotinib[tiab] OR Herceptin[tiab] OR "HER2 inhibitor*"[tiab] OR "HER-2 inhibitor*"[tiab] OR "anti-HER2"[tiab] OR "anti-HER-2"[tiab] OR Pertuzumab[tiab] OR Kadcyla[tiab] OR tarceva[tiab] OR	84,468		

			IRESSA[tiab] OR "HER2-targeted"[tiab] OR "HER-2-targeted"[tiab])			
	#4	Exposure = genome-wide methylation and gene expression profile	("methylation"[Mesh] OR "Epigenesis, Genetic"[Mesh] OR "Epigenomics"[Mesh] OR "Epigenome"[Mesh] OR "CpG Islands"[Mesh] OR "CpG Island*"[tiab] OR epigenome*[tiab] OR epigenetic[tiab] OR epigenetics[tiab] OR epigenomic[tiab] OR epigenomics[tiab] OR methylome[tiab] OR methylomes[tiab] OR methylat*[tiab] OR hypomethylat*[tiab] OR hypermethylat*[tiab] OR "EWAS"[tiab] OR "genome-wide association study"[Mesh] OR "alternative splicing"[Mesh] OR "Polymorphism, Single Nucleotide"[Mesh] OR "gene expression"[Mesh] OR "gene expression profiling"[Mesh] OR "Sequence analysis,	1,628,461		

			RNA"[Mesh] OR "DNA copy number variations"[Mesh] OR genomics[Mesh] OR "whole genome sequencing"[Mesh] OR "alternative splicing"[tiab] OR "single nucleotide polymorphism*"[tiab] OR SNP[tiab] OR SNPs[tiab] OR "gene expression"[tiab] OR miRNA[TIAB] OR microRNA[tiab] OR "RNA sequenc*"[tiab] OR "RNA profiling"[tiab] OR "mRNA-seq"[tiab] OR "copy number variations"[tiab] OR GWAS[tiab] OR "genome wide"[tiab] OR genomic[tiab] OR genomics[tiab] OR "whole-genome sequenc*"[tiab] OR WGS[tiab] OR transcriptome[tiab])			
	#5	Outcome = response to treatment	("treatment outcome"[Mesh] OR "Disease-Free Survival"[Mesh] OR "progression-Free Survival"[Mesh] OR "Drug resistance, Neoplasm"[Mesh] OR	5,723,752		

			"survival analysis"[Mesh] OR prognosis[Mesh] OR "Kaplan-Meier Estimate"[Mesh] OR outcome[tiab] OR survival[tiab] OR "pathological complete response"[tiab] OR pCR[tiab] OR sensitivity[tiab] OR benefit[tiab] OR resistance[tiab] OR recurrence[tiab] OR survivors[tiab] OR predictive[tiab] OR predictor[tiab] OR predictors[tiab] OR prognosis[tiab] OR "Kaplan-Meier"[tiab])			

Supplementary Table 3. Summary characteristics of one study of genome-wide DNA methylation measured in breast cancer tissue and risk of recurrence among HER2-positive, trastuzumab-treated breast cancer patients (*n* = 1)

Study design	**Retrospective study:** Furrer [2022]
Breast cancer patients	**Number of patients:** 12<br>**Mean age:** 51.3 y<br>**Postmenopausal patients:** 67%<br>**ER-positive breast cancer:** 67%
DNA methylation	**Measurement method:** Infinium HumanMethylation450 BeadChip array<br>**Tissue used:** FFPE

	**Tumor cell fraction:** ≥ 70%
Outcome	**DFS**

DFS: Disease free survival; ER: estrogen receptor; FFPE: formalin-fixed, paraffin-embedded; n: number of studies; y: years.

Supplementary Table 4. Summary characteristics of studies of gene expression measured in breast cancer tissue and risk of recurrence among HER2-positive breast cancer patients treated with HER2 targeted therapies (*n* = 9)

Study design	**Retrospective studies:** *n* = 5 (Khoury[1], Gámez-Pozo[2], Triulzi[3], Merry[4], Zhao[5])<br>**Prospective studies:** *n* = 4 (Harris[6], Guarneri[7], Sorokin[8], Perez[9])
Breast cancer patients	**Number of patients:** 9 to 849<br>**Age:** median - 52 to 58 y (*n* = 2) (Gámez-Pozo[2], Triulzi[3]), mean - 55.2 y (*n* = 1) (Sorokin[8]), < 45 y in 60.9% (*n* = 1) (Harris[6]), < 50 y in 48.3% (*n* = 1) (Perez[9]), NR (*n* = 4) (Khoury[1], Merry[4], Guarneri[7], Zhao[5])<br>**Postmenopausal patients:** NR (*n* = 9)<br>**ER-positive breast cancer:** 43.4% to 69.6% (*n* = 3) (Harris[6], Gámez-Pozo[2], Triulzi[3]); none in one study Sorokin[8]); NR in five study (Khoury[1], Guarneri[7], Merry[4], Zhao[5], Perez[9])
Gene expression	**Measurement methods:** HumanHT-12 v3 BeadChip (*n* = 1) (Khoury[1]), HumanHT-12 v4 BeadChip (*n* = 1) (Triulzi[3]), Affymetrix Human Genome U219 array (*n* = 1) (Gámez-Pozo[2]), Affymetrix GeneChip 3' IVT Express kit (*n* = 1) (Guarneri[7]), Illumina HiSeq 2500 platform (*n* = 1) (Merry[4]), Illumina HiSeq 3000 (*n* = 1) (Sorokin[8]), GeneChip U133 Plus 2.0 Gene Array (*n* = 1) (Harris[6]), DASL technology (*n* = 1) (Perez[9]), Agilent UNC Perou Lab Homo sapiens 1X44K Custom Array/Illumina HumanHT-12 WG-DASL V4.0 R2 expression beadchip/Affymetrix technology using the HG-U133 Plus 2.0 GeneChip array (*n* = 1) (Zhao[5])<br>**Tissue used**: fresh frozen (*n* = 5) (Harris[6], Khoury[1], Guarneri[7], Merry[4], Zhao[5]), FFPE (*n* = 4) (Gámez-Pozo[2], Triulzi[3], Sorokin[8], Perez[9])<br>**Tumor cell fraction:** ≥ 70% (*n* = 2) (Triulzi[3], Sorokin[8]), ≥ 80% (*n* = 1)

	(Khoury [1]), ≥ 500 cancer cells per case (*n* = 1) (Harris[6]), NR in five studies (Gámez-Pozo[2], Guarneri[7], Merry[4], Zhao[5], Perez[9])
Outcome	**pCR:** *n* = 5 (Harris[6], Gámez-Pozo[2], Guarneri[7], Merry[4], Zhao[5])<br>**DFS:** *n* = 2 (Khoury[1], Sorokin[8])<br>**RFS:** *n* = 2 (Perez[9], Triulzi[3])

DFS: Disease-free survival; ER: estrogen receptor; FFPE: formalin-fixed, paraffin-embedded; n: number; NR: not reported; pCR: pathologic complete response; RFS: relapse-free survival; y: years.

Supplementary Table 5. Summary characteristics of studies of genome-wide miRNA expression measured in breast cancer tissue and risk of recurrence among HER2-positive, trastuzumab-treated breast cancer patients (*n* = 2)

Study design	**Retrospective studies:** *n* = 2 (Du[10], Ohzawa[11])
Breast cancer patients	**Number of patients:** 14 to 40<br>**Median age:** 55 y (Ohzawa[11]), NR in one study (Du[10])<br>**Postmenopausal patients:** NR<br>**ER-positive breast cancer:** NR<br>**HR-positive breast cancer:** 42.5% to 50%
miRNA expression	**Measurement methods:** Agilent miRNA array (*n* = 1) (Du[10]), Agilent SurePrint G3 Human miRNA microarray (*n* = 1) (Ozhawa[11])<br>**Tissue used:** FFPE (*n* = 2)<br>**Tumor cell fraction:** ≥ 80% (Du[10]), NR in one study (Ohzawa[11])
Outcome	**DFS:** *n* = 1 (Du[10])<br>**pCR:** = 1 (Ohzawa[11])

DFS: Disease free survival; ER: estrogen receptor; HR: hormone receptor; FFPE: formalin-fixed, paraffin-embedded; n: number of studies; NR: not reported; pCR: pathologic complete response; y: years.

Supplementary Table 6. Summary characteristics of one study of genome-wide long intergenic noncoding RNA (lincRNA) expression measured in breast cancer tissue and risk of recurrence among HER2-positive, trastuzumab-treated breast cancer patients (*n* = 1)

Study design	**Retrospective study:** Merry[4]

Breast cancer patients	**Number of patients:** 13<br>**Mean age:** NR<br>**Postmenopausal patients:** NR<br>**ER-positive breast cancer:** NR
lincRNA expression	**Measurement method:** Illumina HiSeq 2500 platform<br>**Tissue used:** fresh frozen<br>**Tumor cell fraction:** NR
Outcome	**pCR**

ER: Estrogen receptor; n: number of studies; NR: not reported; pCR: pathologic complete response.

Supplementary Table 7. Summary characteristics of studies of copy number alterations (CNA) measured in breast cancer tissue and risk of recurrence among HER2-positive breast cancer patients treated with HER2 targeted therapies (*n* = 2)

Study design	**Prospective studies:** *n* = 2 (Guarneri[7], Walsh[12])
Breast cancer patients	**Number of patients:** 11 to 68<br>**Median age:** 54 y for pCR and 50 y for non-pCR (Walsh[12]), NR in one study (Guarneri[7])<br>**Postmenopausal patients:** NR (*n* = 2)<br>**HR-positive breast cancer:** 54.5% (Walsh[12]), NR in one study (Guarneri[7])
CNA	**Measurement method:** Illumina HiSeq (*n* = 1) (Walsh[12]), Affymetrix Genome-wide Human SNP (*n* = 1) (Guarneri[7])<br>**Tissue used:** fresh frozen tumor (*n* = 2) (Guarneri[7], Walsh[12])<br>**Tumor cell fraction:** NR (*n* = 2)
Outcome	**pCR** (*n* = 2)

HR: Hormone receptor; n: number; NR: not reported; pCR: pathologic complete response; y: years.

Supplementary Table 8. Summary characteristics of one study of protein expression measured in blood and risk of recurrence among HER2-positive, trastuzumab-treated breast cancer patients (*n* = 1)

Study design	**Retrospective study:** Yang[13]

Breast cancer patients	**Number of patients:** 6<br>**Median age:** 50 y<br>**Postmenopausal patients:** NR<br>**ER-positive breast cancer:** 33.3%
Protein expression	**Measurement method:** TMT-6 plex Isobaric Label Reagent Set<br>**Tissue used:** blood
Outcome	**pCR**

ER: Estrogen receptor; n: number of studies; NR: not reported; pCR: pathologic complete response; y: years.

Supplementary Table 9. Summary characteristics of studies of somatic or germline mutation profile and risk of recurrence among HER2-positive, trastuzumab-treated breast cancer patients (*n* = 2)

Study design	**Prospective studies:** *n* = 2 (Shi[14], Lesurf[15])
Breast cancer patients	**Number of patients:** 48 to 203<br>**Median age:** 50 y (*n* = 1) (Lesurf[15]), < 65 y in 90.3% (*n* = 1) (Shi[14])<br>**Postmenopausal patients:** NR (*n* = 2)<br>**ER-positive breast cancer:** 48.3% to 58.3%
Polymorphisms analysis	**Measurement method:** Illumina HiSeq 2000 (Shi[14]), whole genome sequencing (WGS) and whole exome sequencing (WES) + Illumina HiSeq 2000 platform (Lesurf[15])<br>**Tissue used:** fresh frozen for somatic mutation (*n* = 2) (Shi[14], Lesurf[15]), blood for germline mutation (*n* = 1)* (Lesurf[15])<br>**Tumor cell fraction:** > 10% (*n* = 1) (Shi[14]), NR in one study
Outcome	**pCR** (*n* = 2)

*One study assessed somatic and germline mutations and used two types of tissue. ER: Estrogen receptor; n: number of studies; NR: not reported; pCR: pathologic complete response; y: years.

Supplementary Table 10. Risk of bias of included studies

Studies	Bias in selection of participants into the study	Bias due to confounding	Bias in measurement of exposure	Bias in measurement of outcomes	Bias due to missing data (selection)	Bias in selection of the reported result	Overall within study risk of bias
Methylation							
Furrer *et al.*, 2022	Low	Moderate	Low	Low	Low	Low	Moderate
Gene expression							
Khoury *et al.*, 2010	Low	Critical	Low	Low	Low	Moderate	Critical
Triulzi *et al.*, 2015	Low	Moderate	Low	Low	Low	Moderate	Moderate
Gámez-Pozo *et al.*, 2014	Moderate	Moderate	Low	Low	Low	Moderate	Moderate
Guarneri *et al.*, 2015	Low	Critical	Low	Low	Low	Moderate	Critical
Merry *et al.*, 2016	No information	Critical	Moderate	Low	Low	Moderate	Critical
Harris *et al.*, 2007	Moderate	Critical	Low	Low	No information	Moderate	Critical
Zhao *et al.*, 2018	No information	Critical	No information	Low	No information	Moderate	Critical
Sorokin *et al.*, 2020	Low	Critical	Moderate	Low	No information	Serious	Critical
Perez *et al.* 2015	Moderate	Moderate	Low	Low	No information	Low	Low
miRNA expression							
Du *et al.*, 2016	No information	Moderate	Serious	Low	Low	Low	Serious
Ohzawa *et al.*, 2017	Low	Critical	Low	Low	No information	Moderate	Critical
lincRNA expression							
Merry *et al.*, 2016	No information	Critical	No information	Low	Low	Low	Critical

Copy number alteration							
Guarneri *et al.*, 2015	Low	Critical	Low	Low	Low	Moderate	Critical
Walsh *et al.*, 2020	No information	Critical	Low	Low	No information	Moderate	Critical
Protein expression							
Yang *et al.*, 2020	Moderate	Moderate	Low	Low	No information	Moderate	Moderate
Somatic or germline mutation profile							
Shi *et al.*, 2017	Low	Low	Low	Low	No information	Low	Low
Lesurf *et al.*, 2017	Low	Critical	Moderate	Low	No information	Low	Critical
Overall across studies risk of bias	No information	Critical	Serious	Low	No information	Serious	

Supplementary Table 11. Genes differentially expressed in breast cancer tissues of cases compared to controls

Gene	Direction change in gene expression in resistant tumors	Reference
HGF, IGF-1, PDGF, PTN, C-MET, LEP-R, PI3K p85, MAP2, SFRP1, p63, BOC	Increased	Harris[6]
ACP2, ACTN4, AGPAT1, AK2, ALDOA, ALG3, AP2A1, AP2M1, APH1A, ARHGDIA, ASNA1, ATP5J2, ATP6V0B, ATXN2L, BACE2, BAT2, BSG, C11orf49, C16orf13, CAPNS1, CDK18, CDK4, CENPB, CHPF, CLPTM1, COPE, COX6C, CPNE1, CSNK1G2, CSNK2B, CSTB, CYC1, DPP7, DSCR3, DUSP3, DYNC1H1, DYRK1A, EFTUD2, EIF5A, EIF5AL1, ENG, ERGIC3, ERICH1, FARSB, FKBP8, GBA, GNB2, GPX4, HIST1H3F, HNRNPL, ITPK1, KDELR1, LSS, LTBP4, MAF1, MED24, MED25, NDRG3, NUCB1, OST4, PCDH1, PHC2, PIGQ, PIGT, PLOD3, POLR2E, PRKACA, PRMT2, PSMB2, PSMD8, PSMF1, PSMG4, PTTG1IP, PYCR2, RAB11B, RNF220, RNPS1, ROD1, RPL18, RPL28, RPS5, SERINC2, SH3GL1, SH3GLB2, SHARPIN, SHISA5, SHKBP1, SLC19A1, SLC25A23, SOD1, SRM, SSNA1, TAF6, TAPBP, TMC6, TMEM54, TP53, TSC22D4, TUBB, UBE2I, VPS28, ABCA3, ABHD12, ABHD14A, ABI1, ACTR1B, AGFG1, AGPAT3, AGTRAP, AHDC1, ALKBH6, ANKS1A, AP2B1, AP3S2, APBA3, ARAF, ARF1, ARF3, ARF5, ARFGAP2, ARHGAP33,	Decreased (n = 424)	Gámez-Pozo[2]

ARPC1A, ARPC1B, ARPC4, ASB16, ASL, ATF6B, ATF6B, TNXB, ATG4B, ATG4D, ATP13A1, ATP5D, ATP5J2, PTCD1, ATP5SL, ATP6V0A1, ATP6V0A2, ATP6V0D1, ATXN7L2, BAT5, BATF3, BCAT2, BCKDK, BCL7B, BCL7C, BOK, BTBD2, C10orf25, C11orf90, C17orf68, C18orf10, C18orf23, C19orf12, C19orf28, C19orf56, C19orf62, C1orf144, C20orf24, C20orf27, C20orf4, C20orf54, C21orf33, C21orf59, C8orf30A, C8orf30B, C9orf7, C9orf89, CALCOCO1, CAMK1D, CARS2, CASP2, CDC23, CDC37, CDIPT, CDK11B, CDK20, CDK2AP2, CDS2, CHST10, CKS1B, CNDP2, COMMD9, CORO7, COX8A, CPNE2, CPNE9, CPSF1, CSK, CUTA, CYB5A, CYTH1, DCTN1, DDB1, DDX39, DENND3, DEXI, DGAT1, DHPS, DIP2A, DNASE1, DNER, DNM2, DOCK6, DPM2, DUSP23, DYNLRB1, EIF2C2, EIF3G, ETHE1, FAM136A, FAM96B, FAM98C, FARP2, FASTK, FBXL19, FIBP, FKBP1A, FKBP1A, FKBP1C, FZR1, G6PC3, GART, GBA, GBAP1, GDI1, GGA2, GNA13, GPAA1, GPR137, GPR89A GPR89B, GPR89C, GRINA, GRK6, HIGD2A, HIP1, HIST1H2AC, HIST1H2BG, HIST1H3D, HIST1H3H, HIST1H3I, HIST1H4E, HIST1H4J, HIST1H4K, HIST1H4K, HIST2H3A, HIST2H3C, HIST2H3D, HLCS, HPS4, IFITM2, IMPDH2, IPO13, IPO5, IRF3, KCNK6, KCTD5, KHSRP, KIAA0146, KIAA0355, KIAA1310, KIAA1522, KIAA2013, KIF12, KIF18B, KIF22, KLHDC3, KRTCAP2, LAMP1, LAPTM4A, LAPTM4B, LAS1L, LETMD1, LGMN, LIN37, LOC100294376, TECR, LRFN3, LYPLA1, MAD2L2, MAEA, MAMDC4, MAN1B1, MANBAL, MANF, MGAT4B, MGLL, MIF, SLC2A11, MLLT1, MOBKL2A, MOSPD3, MPDU1, MPND, MPV17L2, MRPL4, MSI2, MSRB2, MTMR14, NARFL, NCKIPSD, NDUFA11, NDUFA3, NDUFA7, NDUFV3, NINJ1, NOMO1, NOMO2, NOMO3, NOP10, NOTCH4, NR1H2, NUBP2, OAZ1, OLFML2A, OR5L1, OR5L2, OS9, OXSR1, PABPC1, PCBD1, PCMT1, PDK3, PEMT, PFKFB3, PFN2, PGD, PGLS, PIGP, PIGU, PIGZ, PIK3R1, PLEKHH3, PNPLA5, POLD2, POLL, POM121C, POMT1, PPFIA3, PPP1R15A, PPP4C, PPP4R1, PPPDE2, PRKCSH, PRMT1, PRR13, PSENEN, PSME3, PTBP1, PTGR1, PTK2, PTPN23, QTRT1, RAB8A, RANGAP1, RHBDF2, RHEB, RHOG, RNF167, RPL13A, RPL15, RPL18A, RPL37, SNORD72, RPN1, RPS2, RPS26, RPS28, RUVBL2, SEMA3F, SF3B4, SFRS15, SLC25A44, SLC35A4, SLC35C2, SLC35E1, SLC38A7, SLC6A9, SMPD4, SNORA23, SNORA47, SNX24, SPATA13, ST6GALNAC6, STK11IP, STK25, SUPT3H, TAF12, TAGLN2, TARS2, TATDN2, TBCD, TCEA2, TCF25, TECR, TFDP1, TGIF1, TIAM1, TIMM16, TLR7, TMEM109, TMEM120B, TMEM161A, TMEM201, TMEM203, TMEM205, TMEM219, TMPRSS9, TNIP1, TRAPPC1, TRAPPC10, TRIM28, TRIP6, TSEN34, TSG101, TXN, UBA52, UBAP2L,		

UBE2G2, UBE2M, UBR4, ULK4, UROD, USP16, USP25, UTY, VPS72, WASF2, WBP2, WDR90, WDTC1, YIF1B, YIPF2, YIPF3, YWHAG, YWHAZ, ZC3H3, ZDHHC18, ZMAT2, ZNHIT1, ZNRD1		
AHI1, APH1A, ATRX, BAT2L2, CALML4, DDX59, DHRS7, EIF4B, ERICH1, EWSR1, FAM198B, FOXP1, GCC2, GTF2H1, HIVEP2, HNRNPA3, IRAK4, IREB2, KIAA1109, KIAA1432, KLHL8, MIA3, MLL5, MON2, NARG2, NEXN, NUCKS1, OPA3, PHF3, PHTF2, PPCS, PPFIBP1, PPIG, PTEN, PYCR2, RECQL, SDCCAG8, SERBP1, SERPINE2, SFRS2IP, SHOX2, SMNDC1, STK38L, STYX, TCF4, TNRC6B, UBQLN2, USP15, ZBTB1, ZBTB43, ZFAND5, ZNF226, ZNF638, ZNF766, MARCHF6, AASDHPPT, ABCB10, ACBD3, ACPP, ACTG2, ACTR2, ACVR2A, ADAM3A, ADAMTS5, ADO, AKR1D1, AKT3, ALPK2, AMMECR1, ANAPC4, ANKRD13C, ANKRD32, ARAP2, ARFGAP3, ARHGAP18, ARID1B, ARID4A, ARL1, ARL6IP5, ARNT, ARRDC4, ASAH1, ASXL2, ATAD1, ATF4, ATG2B, ATP5I, ATXN3, BAG5, BAGE, BAZ2B, BCAP29, BCL2L2, BCL6, BCLAF1, BDNF, BLNK, C10orf119, C10orf4, C12orf11, C12orf24, C14orf145, C15orf40, C1orf156, C1orf25, C1R, C4orf34, C5orf24, C5orf44, C5orf53, C7orf41, CACHD1, CAMK2D, CASC4, CASK, CATSPER2, CCDC14, CCDC76, CCDC88A, CCNL1, CCPG1, CD302, CD48, CD99L2, CDON, CEP57, CEPT1, CHD3, CIR1, CLINT1, CLIP1, CLK3, CMAH, CNIH4, COL4A3BP, CORIN, CORO1C, CREB1, CSDE1, CSNK1G3, CSTF3, CTBP1, CTDSPL2, CYP3A5, DARS, DDHD1, DDX3X, DEGS1, DIDO1, DNAJC13, DNAJC24, DPYSL3, EIF2AK4, EIF4E, EIF4ENIF1, EIF5, EIF5B, ENAH, EPHX4, ERMP1, ETNK1, EXOSC2, FAIM, FAM115A, FAM168B, FAM46A, FAM63B, FAM71F1, FAM83C, FAM98A, FBLN1, FBXO11, FBXO22, FBXW2, FBXW7, FCF1, FDX1, FGFR1OP2, FIP1L1, FKBP3, FLCN, FNIP1, FOSL2, FOXJ2, FOXRED2, FRYL, FYTTD1, GATA2, GGCX, GLIS3, GLS, GLTSCR2, GNAQ, GNPAT, GOLGA2, GOLGA7, GORAB, GPR155, GRB10, GREM2, GUCY1A3, HAUS3, HBS1L, HERPUD2, HIVEP1, HNRNPA0, HNRNPC, IBTK, IGF2, INS-IGF2, IL6R, ING3, INTS6, IQGAP2, ITIH5, ITPRIPL2, KDM2A, KIAA1586, KLF3, LAMC1, LARP1, LLPH, LMAN1, LOC440917, YWHAE, LONP2, LRCH2, LRP10, LRRC17, LRRC58, LRRK2, LUC7L2, LYSMD3, MAF, MAP4K5, MED21, MEP1B, MESDC2, MIER1, MKI67IP, MLL2, MMAA, MORF4L1, MOSPD1, MTIF2, MTMR1, MTR, N4BP2, NAA50, NAP1L1, NFIB, NIPBL, NOL8, NSL1, ORMDL2, OSBPL5, OTUD4, OTUD5, PARP11, PBRM1, PCF11, PDCD7, PDPK1, PGBD2, PGM3, PHF21A, PHKB, PI4K2B, PIK3CG, PLEKHA5, POC1B, POLR3G, PPP1R12A, PPP2R5E, PPTC7, PRIM2, PRKAA1, PRKD3, PRPF39, PTP4A1, PTPDC1,	Increased (n = 425)	Gámez-Pozo[2]

RAB1A, RAB3GAP1, RAB3GAP2, RAPGEF6, RBM16, RBM25, RBM34, RBMS1, RBMS2, RCHY1, RCOR3, RECK, RFTN2, RGL1, RHOQ, RICTOR, RLF, RNF149, RORA, RPAIN, RPL32, RPL9, RRAS2, RSF1, RSRC2, RWDD3, SACS, SCRN3, SCUBE3, SDCCAG1, SEC24A, SECISBP2L, SELT, SFRS12, SFRS13A, SFRS13B, SFRS3, SFRS5, SGK269, SHOC2, SIK2, SIPA1L2, SKI, SLAIN2, SLC25A46, SLC30A5, SLC30A7, SLC36A1, SLC37A4, SLC39A14, SLC3A1, SLTM, SMC3, SMEK2, SMU1, SNAP23, SNAPC4, SNX13, SOS2, SPTLC2, ST3GAL2, STAMBP, STEAP2, SWAP70, SYNE2, SYNJ2BP, TAF1, TAF1A, TAF7, TAGLN, TARSL2, TATDN3, TAX1BP1, TBC1D15, TBCEL, TBRG1, TCF12, TFCP2, THBS2, THOC2, THRB, TIAL1, TIRAP, TLK1, TM4SF20, TMCC3, TMEM167A, TMEM200C, TMF1, TMX3, TNFRSF19, TNRC6A, TOR1AIP1, TPSAB1, TPSB2, TRD@, TRIM24 , TRIM34, TRIM34, TRIM6-TRIM34, TRIM38, TROVE2, TSC22D2, TSEN15, TSHZ3, TTC21B, TWIST1, U2AF2, UBA5, UBE2Q2, UBE3A, UGT2A1, UGT2A2, USH1C, USP3, USP46, USP8, UTP15, VDR, WDR26, WDR44, WDR73, WSB1, XIAP, YAP1, YTHDC2, YWHAH, YY1AP1, ZBTB38, ZCCHC2, ZFP14, ZFP91, ZHX1, ZNF148, ZNF167, ZNF189, ZNF22, ZNF234, ZNF268, ZNF280D, ZNF330, ZNF333, ZNF449, ZNF451, ZNF673, ZNF674, ZNF678, ZNF713, ZRANB1		
PROM1, IRX2, SFRP1, SLPI, RBP1, CBS, AKR1C3, HBA2, BEX2, SELM, ZDHHC8, HS.143018, ZNF462, SNORD13, TMPRSS2, HS.193557, HS.193767, TIMP1	Increased (*n* = 18)	Khoury[1]
TRIM37, HIST1H2AE, C3ORF14, FAM84B, UTP18, DCK, NXPH1, LFNG, ZNF239, H2AFJ, GCHFR, PLA2G7, GHR, CKS2, XYLT2, EPN3, ZNF296, NCAM2, RPL29, LACTB2, NDST4, SLC35B1, HUS1B, ADORA2A, NME1, NME1-NME2, SH3BGRL, MRPL27, SYNPO2 L, SPATA20, NETO2, HSPA2, NQO1, ASCL2, EXOC2, MYCN, HEBP1, PHB, CNTNAP2, C9orf152, MIPEP, KIAA1467, TOB1, AKR7A3, SLC12A2, CEACAM6	Decreased (*n* = 46)	Khoury[1]
LTF, LPCAT1, RAB26, C1orf64, KIAA0114, CNTNAP2, CLGN, MGC29506, CNTNAP2, TLR5, MGC29506, LSM7, GALNT7, ARSD, NBEAL2, PNMT, GALNT7, NCOA7, CYP4X1, LFNG, GATM, DNAJC9, FAM84B, XDH, GRB7, TNFSF10, LOC644246, AQP5, GATM, SLPI, RHOC, SPDEF, SECTM1, TNFSF10, MALL, SPDEF, F8, PLA2G16, CAMK2N1, HMG20B, HMG20B, C4orf7, PABPC1L, FGFR4, HSD17B2, EPS8L1, RCAN2, OAZ3, OLFM4	Not reported (*n* = 49)	Guarneri[7]
ERBB2, GRB7, ORMDL3	Decreased (*n* = 3)	Triulzi[3]
C1orf186, ESR1, RERG	Increased (*n* = 3)	Triulzi[3]
C14orf173, C17orf173, C1QTNF7, C2orf48, CDK5R1, CPA3, CPNE5, CTSG, DBN1, DERL3, GPC2, HIST1H2BM, IGFN1, INTS5, KPNA5, LRRC8D, MANF,	Not reported (*n* = 35)	Triulzi[3]

MEX3A, MS4A2, ODC1, PGAP3, PLEKHA8, PRDM1, PSMD3, RGS13, RIPK4, SLC18A2, SLC8A3, SOX11, TCAP, TNFRSF17, TPSAB1, TPSG1, VPS37B, WASH2P		
RPS3A, PSMD3, MUC6, RPL41, HCAR1, PHB, GSDMB, GOSR1, MYH11, RPL7, KYNU, NME1, MYC, ICT1, EEF1B2, LRRC37BP1, PEG10, NPM1, MAOB, CSTA, HAUS8, HIST1H2AI, PALM3, SFRP1, TOB1, NTRK2, S100B, MYO18A, SCARNA13, EEF1A1, CPD, SELL, GABRP, ID4, GIT1, PIP, SYNM, GNLY, DEPTOR, JAM2, CXCL9, MEG3, POF1B, PABPC1, RPL10, BLMH, ITGB6, CD36, KIAA0100, RPL6, AIM1, GDPD1, HIST1H2BG, PLA2G4A, NME2, ZNF652, GPX3, UTP18, TLCD1, TMEM86A, PPARG, GATM, KAT7, RPS7, FLOT2, MATN2, RBP7, NUFIP2, HNRNPUL2-BSCL2, MME, KLRK1, RPS27, G0S2, RPPH1, LOC541471, CLU, MBTD1, TXNDC5, AP1G2, LOC100132707, PFN1, SLC35B1, SPAG9, PRR13, LDHB, AKAP1, RPL17, ETFB, BCAT1, EIF3H, SNF8, LRRC59, SNHG3, ACSL5, VCAM1, PPP1R9B, ATP8A1, ARHGAP10, VLDLR, SH3D19, CSNK2A1P, TPD52L1, HLA-DMA, NSRP1, GZMK, MIR205HG, CYTIP, PPIA, SYNPO2, NUP93, NEFH, CD3E, EIF3E, PELP1, CDK5RAP3, S1PR1, CGNL1, TRIP10, OXCT1, NPIP, PADI2, SMG8, NPPA-AS1, CCDC11, FAIM3, KIAA0922, ARHGAP26, FHL1, PTN, PDGFA, MPP6, ORMDL3, PPAT, CLK2, F13A1, TOM1L1, GIMAP7, DMD, SCARNA5, AHSA2, PSMC1, TFRC, SPOP, SSH2, NDUFAF2, GGA3, NUP85, SORBS1, SUPT6H, CHI3L1, C17orf80, GNAI1, ARHGEF6, PHF12, FLJ35776, CPED1, AQP3, MEST	Decreased (*n* = 158)	Merry[4]
TMEM107, STAT2, TXNDC16, PCDHGB6, ZNF281, WBP2, ST7-OT4, IFT122, HLA-F-AS1, ACTR1B, ADAL, TMCO1, HMGN3, STT3A, GPATCH2, GSR, RFWD2, MCF2L, MTUS1, DNAAF2, GXYLT1, USP19, NFIA, PSMA1, GCNT1, CCDC71L, TMEM25, B3GALNT2, PCDHGA5, SPOCK1, ANKRD52, BUB3, C21orf33, KIAA1377, PBX2, IDH3B, GPD2, HN1L, ZNF746, MEA1, NSUN3, C1orf38, PIAS3, RAB21, GNPDA1, BRD1, DSTYK, RNF146, CAPG, SAP30L, NEU1, PIM2, NSL1, CAPN2, TSTD1, ENAH, CCDC56, GALNT5, FAM165B, NACC1, CRELD2, FASN, NDUFB1, POLR3F, ZSWIM6, TBC1D9, NAV1, CYSTM1, DENND1B, ABHD2, GPR137B, SMUG1, ATG12, ZNF75D, VHL, PON2, RDH11, ZBTB8OS, CPNE1, TMEM128, ZNF681, LCA5L, CRLS1, TFG, NFKBIA, TCN1, CDK1, PARL, HSD17B6, UBE2Q2, MEPCE, SAA1, CAMSAP2, CLSTN3, CXorf21, EXTL2, ADAR, C1orf210, MOSPD3, AMIGO2, MRPL9, CLDN12, CCDC104, VPS28, C14orf2, GPR160, TIPARP, NUDT3, C10orf35, RPL36A, RABL2B, CDK2, ZNF669, DSTN, DNAJC14, STOML2, PROM2, FAM129A, ANKRD5, PCID2, SSU72, KIAA0101, HDAC7, WDR78,	Increased (*n* = 1383)	Merry[4]

GNPAT, UPRT, NDUFA1, MOK, MARCKS, DDIT3, ZNF577, SLC41A2, MLLT1, C3orf23, GPR56, SEC61B, ACBD6, RNF219, TTPAL, ATOX1, EPRS, PARP1, ZCCHC10, SNORA50, OSGEP, CMPK1, PDGFRL, POFUT2, THBS2, GLT8D2, DDX60L, PPP1R2, TMED7, PAIP1, UBE2F, LYRM5, CBR1, CLEC4A, ZNF92, RNF11, PRAF2, FASTKD3, ZNF738, EIF1B, ARPC1A, GCA, P4HA2, PGK1, RPL35A, TMEM242, CMAS, C12orf26, HSD17B7, SEC13, TBCEL, BPGM, N6AMT2, TCEB1, PLAUR, AGAP3, KITLG, C1orf144, IRAK3, TRAF5, CASP1, PCDHGB8P, AMACR, COMMD5, TCF19, C20orf3, TOMM7, UQCC, FLJ27352, TCEB2, CCND1, UQCRHL, TTC39A, MIR22HG, NT5C3, TSR3, PSMB3, ZFAND6, LYSMD3, IGSF3, PLEKHS1, TSEN34, C4orf32, RRBP1, FAM63A, EMB, AIDA, PTP4A2, CEACAM5, PPP3R1, FAM104B, TPBG, HSPA13, KRAS, UBL4A, HMGXB4, PDIA4, DCTN6, MAN2A1, GABARAPL2, GPR107, CHMP1B, INSIG1, SP100, FIS1, VIMP, COL8A1, ACAD9, SPRTN, RFK, FNTA, KIAA2013, ACACA, KIAA0930, RGS2, NPRL2, HMGN5, MARCHF1, GSTP1, KRTCAP2, MRPS24, HSP90AB2P, BROX, PAM, PHF2, SPINT2, FASTKD5, HPRT1, SPP1, SLC24A3, NMI, PTGFRN, CXCL17, PLAT, KIF13A, FBP1, FFAR2, WRB, PDIA5, RPIA, SRPX2, TNFAIP6, UBFD1, VMP1, LOC400043, LOC100133991, KDELC1, CDCA2, ARMC10, EXOSC4, TM2D3, GLB1, HOXD8, SF3B5, GJA1, POP4, BPNT1, MRPL41, TSR2, TRAPPC3, NRAS, VAPB, PIP4K2B, NUF2, SIX1, UBE2A, FAM46A, RPS27A, GPC6, C20orf20, CD63, NDUFC2, UHMK1, FAM100B, COL5A1, MRPS18B, WDR26, CAPN8, C6orf62, CACUL1, ISOC1, CYB5R1, TCTEX1D2, TMED9, TMEM41B, HRAS, LOC100134229, FAM168A, HDGFRP3, MEF2BNB, TNIK, C21orf88, MACC1, DEDD, ATP6V0C, GADD45GIP1, DTWD2, REEP3, PPP2R2A, NRIP1, MYCL1, TMSB4X, SEC22B, SIKE1, DYX1C1-CCPG1, EEF1A2, ZNF439, C6orf47, TSPAN15, HTRA1, TBL1XR1, ANKRD42, ZNF678, JMJD7, CA12, C17orf108, TEAD4, SLC25A38, BCDIN3D, NBPF1, SLC25A26, PPP1R3C, RNF181, SNX7, SSX2IP, ARPC5L, C20orf24, ANGEL2, DHFR, LUM, ADIPOR1, TRIL, NAA20, PEX7, COX7B, PIR, CAPZB, MSX2, CRIP2, UBE2D2, MRPL15, RNASEH2A, ENSA, CD2AP, MYBL2, PSMD11, SIK1, AKIRIN2, PRSS16, ENPP4, UBE2B, C18orf32, FAM127C, C3orf78, SOX4, ZCRB1, RAB38, LYST, ATP6V1F, AKAP5, COMMD6, GUK1, GAL3ST4, GPX1, RRN3, DDX60, TSTA3, PSMA7, PLEKHG3, SMPDL3B, SCAMP3, PCDHGA10, TSEN15, PCDHB5, GRINA, RBX1, B4GALT1, RPL39, ZNF674, LMNA, FBXL20, LOC375190, SEC11A, SLC25A24, JUN, PSMA4, CBX4, PLAC2, R3HDM2, PERP, TM7SF3, MRPS21, RNF2, SNAPIN, CNIH4, RIN2, TIPRL, CPE, TAX1BP3, BCL9, ZNF362, PSMD13, ZNF768, HLA-C,		

SLC25A44, U2AF1, MFSD5, SPCS1, SLC35D1, NUP133, SRXN1, TGIF2, AKT1, GADD45B, TMEM51, CSRNP2, OCLM, MT1F, SLC25A37, SLC9A8, RPLP1, PLCE1, FRAT2, ZWINT, TAF5L, AKIRIN2-AS1, FAM32A, HSPB1, TCTA, CSTB, TBC1D8B, SLC44A1, OMD, GMNN, MMS19, DYNLT1, CRYZ, NDUFA13, SNORA33, HDAC1, TPI1, NQO1, WIBG, ZNF329, HMCN1, TSFM, DACH1, LOC100507217, MBOAT2, SLC19A2, GSN, C6orf211, AKR7L, FAM114A1, CLDN7, YOD1, RNF24, SUMO3, TOX3, SLC35A1, SAP30, CDKN2B, PSMG3, PAQR7, GOLM1, MOCS2, ATP6V0B, COL10A1, DOK1, ZNF883, ESRRA, COL3A1, C12orf32, SMYD2, POLB, CREB3L1, GOLGA2, PLAC8L1, COPZ2, ENPP1, STMN1, PRRG4, ZBTB2, ZSWIM1, PPP2R5A, ARMCX5, RTP4, FST, C12orf23, LOC100303749, ID2, ABT1, ASAH1, C1orf31, FAM105A, LOC550643, SELT, NDUFAB1, SFXN4, STK35, PREB, HERC5, TRAPPC2P1, ARFGAP3, SRA1, HIST1H2AD, WNT7B, KDELR2, GPX7, NABP2, ZCWPW2, CASP7, TOR1AIP2, USF2, TUBD1, VGLL3, ZNHIT3, C6orf225, STX6, TSPAN1, OMA1, CBX7, COL5A2, ISCU, TRUB1, RUNX1, PCBD2, ACPL2, LOX, ALG14, MLLT6, PIN4, CTNNBL1, SLC35B2, ROR2, PCDHGB3, RBM45, CACYBP, PARD6B, ATRN, MAVS, COX20, GTF2H5, SUB1, MAMLD1, TATDN3, MOCS3, WWP2, KAT6B, NDUFS5, LSM1, PYGO2, AHCY, DYNLT3, NDUFS2, SYTL4, NUDT16L1, COMMD3-BMI1, NUPR1, DBI, MLPH, PRKAG1, MARCH5, HIST1H2BD, CREB3, C1RL, EXOSC1, GFPT1, ANGPTL1, DPY19L1, ZNF649, FAM162A, MRPL20, GPR52, IFIH1, CA8, EMC9, AGPAT1, TTC9, TCEAL8, EMD, MMP2, HIST1H4I, C7orf59, TRAPPC6A, CAPN1, FAM198A, ARID4B, FAM151B, DAZAP1, RARA, EMC8, FAM102A, IGFBP5, TMEM19, NHP2, DBNDD1, ENTPD7, C1orf131, CD9, SLC29A1, FBLN1, OCIAD1, FRG1B, FAM118B, RPS29, LINC00467, MPLKIP, RPS12, FIBIN, PRRC1, SNRPC, RNPEP, IDNK, WIPI1, NDUFB11, EEF1D, PBX1, DYNLRB2, MB, ATP9A, SGPP1, REEP1, AATF, ARV1, ZNF720, AKIP1, EXOC8, CCNC, C16orf80, RAP2C, FAM83H, TFPT, FRK, H1F0, ARL8A, ISLR, EFCAB2, IFIT3, UBTD2, GALNT2, VKORC1L1, REPS2, TIMM10, LMCD1, MGST3, CABYR, AJUBA, SUMF1, RPL39L, EPS8L1, C10orf116, ZFP64, ARF1, PAX9, RGS4, SPA17, RAD51, MOSPD1, SLC1A5, SFXN1, KIFAP3, PPCS, LRRC47, TADA2A, TFB2M, STK25, MBD6, IPMK, TMEM158, C1GALT1C1, SH3BGRL, ZNF69, APAF1, HEY2, SRP9, CDK15, H2AFY, KIF1C, RPL37A, PMVK, DSN1, AP1M2, C12orf57, NUCKS1, C1orf9, MEIS3P1, CNN2, FOLR1, TNC, DPM3, PRDX6, C1QTNF3, SLC22A5, PRDX5, BLOC1S2, KIAA1383, TMBIM4, PFDN6, SNURF, MFSD12, PGBD2, EPB41L1, SLC2A10, ATP6AP1, TMEM110, S100A1, C4orf33, PMAIP1, AMZ2,		

AKT2, LIN9, ACP2, ACBD3, ANKRA2, MGC2752, ZNF740, PSMB1, KDELR3, ZNFX1-AS1, NFU1, CLDN4, LOC730102, YPEL3, ISG20, ADCK2, METTL18, APOL2, MAN2B2, SDF2L1, FASTK, LOC100506100, GRHPR, NXPE3, RCC2, CCDC23, ZNF440, PUS7L, CILP, OAT, HNRNPU-AS1, KDELR1, SPR, SLC41A1, B2M, FRS2, RPL19, DRAM2, IGJ, RNFT1, TIMMDC1, STXBP2, COX14, CCDC115, COX16, BLVRA, LOC644656, CCDC167, HCG26, TIMM17A, ORMDL2, MEX3A, ARHGEF2, C20orf11, LOC145837, PSMD4, RAB4A, MRPL52, QSOX1, ARHGEF10L, C16orf13, IFNGR2, SNRPB, NDUFA4, TSPAN13, CTSF, NFIC, GBP3, MAML1, C11orf52, FAM20B, BCAS3, TRMT1L, ATPIF1, ASAP3, ZNF626, RASSF1, XAF1, SATB2, ARPC5, SLC9A3R2, PMEPA1, BMP4, TMSB10, S100A6, GRP, KDM5B, TMEM204, HOXB2, ACTG1, KLHL12, PCDHB10, OLFML3, SPATA17, AMMECR1, SCD5, HDHD1, RARRES3, NGFRAP1, MORF4L2, OTUD7B, DNM3OS, NINJ1, COMMD10, SELENBP1, MAP3K9, GJB2, OLFML2B, TNFRSF19, CRIP1, OAS1, PSMB9, RALGDS, OAZ1, MORN2, MCAM, GPX8, PCDHGB5, AES, GNG5, ZNF552, ZIK1, CARHSP1, RBM14-RBM4, SIRT5, MAGEH1, PPP2R3A, PLSCR1, PPIL1, RPS6KC1, SELK, C5orf43, NDUFB3, ZNF300, PCDHB13, USMG5, SAMD9, CTDSP1, GPR98, PCDHB14, WDR54, ARL6, C16orf53, ARF4, SERTAD4, ABHD16A, MST4, TRIM8, RHBDL2, FAM134B, SNRPF, POFUT1, EFNA1, EMC10, DLGAP1, HIGD1A, SMYD3, BTBD9, MRPL33, TMEM14A, ZSCAN18, SSR4, SIX4, B3GAT3, DNAL4, BAX, TMTC4, PPP1R3B, AMOT, PIGF, LGALS3BP, LOC113230, LOC100506686, NEGR1, ARMCX3, COL1A1, HOXC9, KRT10, SOX9, HOXC4, SNHG8, IRF2BP2, LAMP1, COX7A2, NEDD8-MDP1, TM2D1, SFN, PCCA-AS1, FAM110A, SCARNA3, PRKACA, TUSC2, DDX58, RBP1, ZBTB41, ADRM1, FZD3, LSM2, TUBB4B, KLHL22, MAFB, ZNF486, C1QA, UBE2E1, IER3IP1, C19orf66, DDR1, C7orf73, OST4, SLC4A2, TUBA1C, UBE2L6, SLC37A1, COX17, VASP, VTCN1, ATP5E, DHCR24, FGFR2, RABAC1, ST6GAL2, MZT1, TMEM219, MYEOV2, SEPT8, GLIS2, LGALS8, FOSL2, NHLRC1, KCTD3, TRAPPC2L, HLA-DRB5, CST3, DESI2, SLC24A5, OLFM2, LGALS3, CLEC14A, PBXIP1, EDEM3, SNORA8, ST6GALNAC6, CMIP, PHOSPHO2-KLHL23, CERS2, PVRL2, TMEM218, CDK5R1, HOXC6, TGFB2, PLIN3, ZC3HC1, PSD3, UBXN10, IFITM1, RSAD2, IRX5, EMC6, OGN, MEAF6, LOC100506233, MYO1D, COX7A1, LAGE3, ARMCX2, PRSS23, IKZF3, EMX2, NOL3, TACSTD2, TNFRSF25, PTS, WISP1, ERBB3, MANF, ASPN, PYCR2, WBP5, LGALS9, COL8A2, LRRC15, SAC3D1, GNG11, CHMP4B, POLR2L, MAPKAPK2, EIF2AK2, MUTED-TXNDC5, PEX11A, COMMD3, DCTPP1,		

TIMP2, RGS10, PFKFB3, SCGB2A2, CCDC170, HLA-F, TEAD2, ZNF395, GXYLT2, WSB1, F11R, PTPLAD2, CTSD, ASRGL1, RET, CMTM8, MARCKSL1, ASB13, ABRACL, SERPINH1, C1orf133, FAM108C1, PFDN5, LYPD6, CNPY2, PPAPDC1B, VAV3, ZNF217, RSPH1, HLA-B, WDR83, MAGED1, IL18, C1orf151-NBL1, ANKRD39, MIDN, FLVCR1, LYSMD2, PPAPDC1A, F2RL2, ITGB5, FKBP9L, AGPAT4, CAPS, CMTM3, GATA3, LAMTOR2, IGF1R, IFITM3, FEN1, SULT1A1, RPS21, CXXC5, DUSP14, HS3ST1, TTYH3, BSDC1, MDK, TMEM208, RPS6KB2, CDR2L, DLEU1, IRF9, ATP5L, MYB, CISH, SNRPG, NFKBIZ, KHSRP, COL1A2, CDSN, NPLOC4, PGR, LRRC42, DALRD3, TUBB3, TMC4, CANT1, SLC35A2, TROVE2, TCEA3, SCG5, TSNAX, NDNF, IFI44L, C2orf76, FBXO22-AS1, IFI27L2, TFAP2C, C1orf192, HOXC8, WNT5A, DGCR2, NFKBIL1, CRNDE, PFDN4, CELSR1, AHDC1, TRPT1, LYPLAL1, RWDD3, FLJ22184, SAA2, PPIF, MAFG, SIVA1, NDST1, CMYA5, TLR5, LOC100506730, LRRC31, LOC283335, LFNG, TMEM120A, STK39, SCGB2A1, NDUFA2, EPHB4, CDCA4, C10orf47, PRKACB, COL6A1, ZNF428, RAI2, OAS2, LEAP2, ROMO1, GREB1, SNORD97, TMEM45A, OAS3, YRDC, B3GALT5, XBP1, CHTF8, ARHGDIA, PPM1K, DUSP23, SCUBE2, PQLC3, JUND, P4HB, SLC39A6, NDUFB4, CAMK2N1, S100A14, CA2, FOXA1, TMEM141, DBNDD2, KCNK1, ARMCX1, PPIC, S100P, PSEN2, GALNT6, NPTXR, PARP9, MAGED2, FLJ44635, TP53TG1, UQCRQ, C17orf61, MZT2B, IFIT5, MESDC1, GNB2, OCEL1, PIGX, HES1, NTPCR, PQLC1, H3F3AP4, PDF, PKIB, RNF187, CTHRC1, SDC4, TXN, BRP44, LOC648987, NDUFA3, MUC1, DPM1, CYP4X1, LOC284215, USP32, FBXO16, PLA2G16, RNF122, STK36, CEBPB, TMEM9, TCEAL1, BBC3, ARPC1B, HINT2, APPBP2, ATP5I, S100A8, RTN1, EFNA4, NOS1AP, CLEC3A, ASAH2B, SNORA19, RBM11, TBCC, ARL3, ANXA9, SLC44A4, NR2F6, S100A13, ARF5, EFR3B, OVCA2, PRPS1, VASH2, AAMP, PCSK6, ZBTB22, RPA3, ALYREF, PTPRT, MX2, TMEM106C, CYP2B7P1, RALGPS2, GLRX2, DTX3, GREB1L, MRPL55, NKX3-1, IRX3, SNORA45, PCDHB16, SLPI, GGPS1, GLUL, SLC50A1, C1orf204, PDLIM2, LMAN2, STARD13-AS2, DCXR, SLC39A7, PPDPF, SLC7A2, TMEM98, PTPRU, ELOVL2, ARNT2, UBE2J2, NR2F2, HERC6, TRIM11, MBOAT1, TCEAL4, AGRN, SNORA5A, PCDHGA11, STK16, LY6E, LASP1, ADCY1, SOX12, SPON2, DCAF10, RORC, CACNB3, SNORA27, GFRA1, MACC1-AS1, C1orf64, COL4A5, POC1B-GALNT4, CMPK2, MEX3D, NPM3, IFI44, HNF4G, LOC389493, SLMO2-ATP5E, MX1, C1orf88, FAM125A, HHAT, ZNF703, PCGF2, SNORA6, BST2, SLC4A8, ENPP5, HGD, DDAH2, AMPH, SHISA2, SCCPDH, TMEM161B-AS1,		

SLC24A2, TCEAL3, AGR3, ANAPC11, LOC440335, ARL16, FLJ23867, KRTCAP3, CFB, TMEM125, RNU4ATAC, SERPINA3, C1orf226, KLF4, ZNF238, PI3, C19orf33, STRA13, SNORA57, SNORD99, AGR2, CEBPD, SERPINA5, BCAS4, ESR1, FAM195B, IGFBP2, INO80B, MUC5B, MIR4263, IFIT1, MIR558, SNORA61, PRR15, IFI6, TFF1, TFF3, GRB14, MIR4296, TRIM58, NAT1, ASCL2, SNORA21, SNORA25, WFDC2, IFI27, CAPN9, PYCR1, SNORD111, ZG16B, ISG15, BMPR1B, CLIC6, SHROOM1, GDF9, MIR320B2, MIR3662, SNORA32, SNORA70E		
NR for individual analysis, no overlapping	Increased : GSE22358 (*n* = 488) GSE62327 (*n* = 548) GSE66305 (*n* = 105)	Zhao[5]
NR for individual analysis, SOX11, ATP8B2 and ODF2L in all three datasets	Decreased : GSE22358 (*n* = 493) GSE62327 (*n* = 424) GSE66305 (*n* = 450)	Zhao[5]
REPS1, DRAP1, ZNF697, RSPRY1, EPS15, MRPL50, COPS8, VPS26A, UBA3, BCL2L11, TBCA, SAT2, STX7, ARHGAP18, GLIPR1, CWC15, IRAK3, SNRPD3, MSANTD2, NAPEPLD, YWHAQ, MAP1LC3B, PLP2, SPACA6, ACTR6, SAP18, HNRNPH2, FAM117B, SYPL1, NOC3L, STXBP4, B3GALNT1, ENDOD1, NDUFAB1, FGL2, FAM213A, ARL6IP5, RAB28, ATP5I, NPC2, EPHA4, HS2ST1, EED, STT3A, ARPC1A, RNF214, TMOD2, SMC5, ARNTL2, UST, PPP3CB, PEX3, NT5DC1, TXN, SIKE1, SRP54, NMT2, IPMK, C9orf85, KCTD6, FOXO4, PNPLA8, CHPT1, HTATSF1, CCDC28A, CHORDC1, CADM4, LRIF1, RNF5, MMRN2, C16orf87, FAM217B, DCXR, RASGRP1, CXorf38, MMD, AK9, TOM1L1, SRP9, FZD1, C21orf91, LSM3, PRKAA2, USP14, ITIH5, ISCA1, CYYR1, C9orf64, ADAT2, WASHC3, LPL, COMMD10, NDUFA5, GPD1L, SNORA12, ALYREF, FIG4, SLC39A8, GAPT, PDIK1L, TMEM206, HSDL2, SLC12A2, ATG5, IKZF3, SNRPE, CD164, RNGTT, SESN3, ADGRG6, LYVE1, HBS1L, HMCN1, BMT2, CEP57L1, C5orf30, MCTS1, IMMP2L, ARL13B, RHOBTB3, CLIC5, CPXM1, FAM76B, ABRACL, BAG4,	Decreased (*n* = 402)	Sorokin[8]

PPP3CA, KIF11, CENPE, NR5A2, CLEC14A, SNX3, DPM3, SNHG19, FKBP7, ING3, FRK, NIPSNAP3A, NUDT12, ADAM22, NEK2, TSPAN33, RGCC, RWDD1, PRKAR2B, PDGFRL, HIST1H4C, SMAD9, PLA2G2A, HSPD1P11, ALMS1-IT1, PARM1, ZCCHC17, GABRE, PPP1R1A, THEMIS, COMMD6, FAM184A, MORN2, BMI1, PCDHB3, ZNF132, EEF1A1P1, SNRPG, HIST1H2BK, LRR1, NOV, ALDOC, CDK15, EPHB3, SERPINB8, ARL3, CASP1, FRZB, TSPAN7, AAMDC, CCDC150, ZYG11A, LY96, GATS, GLYATL2, CYSLTR2, MEGF9, KIF20B, TUSC1, IL17RB, CTLA4, GON7, HIST1H4B, AADAT, SEMA5A-AS1, TMEM60, SBSPON, PRKCQ-AS1, ADM, CETN3, IMPA2, TMEM150C, TMEM178B, COX10, DPY19L2, SLC1A2, FOLR2, SLC4A4, FAM155A, SNORA53, DCAF4L1, SLC16A10, TENM2, NDUFAF4, RN7SKP299, SIX1, ST8SIA6, RPP40, ZNF257, MAOA, ST6GALNAC3, HMGN5, TBPL1, PCBP3, EIF3C, NGFR, CD24, HES6, PELI2, PRRG3, MMP12, LDHAL6A, BAALC, ELOVL7, ABCD2, CCNE1, FCN1, IGSF10, SNORA38, CKS2, GNE, RPF2, CLEC10A, RTKN2, RNF144A-AS1, AFF3, SEPT3, DNAJC6, ADCY5, MPIG6B, DMC1, SNORA49, BCL11A, SCARNA1, LAMC2, IGHV3-13, HIST1H2AJ, ADIPOQ, HIST1H3B, CARD8-AS1, CP, SCG2, SULT1C4, SCN9A, NETO2, HSPB6, PTCHD1, CXCL2, RNVU1-15, CDKN1C, NEXMIF, LINC01473, LSM12, MYOT, ATP8A2, TNIP3, LGR6, RBP4, LRRC4C, CIART, MEOX1, MTND1P9, SLC35E4, MT1E, IGHV1-58, MTND4LP30, TOX, RAB11B-AS1, SNORA20, GLDC, NAGS, TMOD1, KIF15, RBBP8P1, SEC14L5, IGHV3-74, ZNF667-AS1, ARL4D, HDAC2-AS2, LIFR-AS1, GLRA3, B3GAT1, TRNP1, SYF2P2, CHST9, RNU7-20P, IGHV4-61, RAB39A, SLC6A14, NKAIN1, BNIP3P10, ANKRD20A5P, BOLA2P2, OR52N4, PLAC8, CENPCP1, LUZP2, TDRD9, PRDM16, PHACTR3, KERA, MRAP2, LINC01139, FOXA3, PLP1, COLCA1, RN7SL434P, TMEM101, LINC00161, LINC02202, CRTAC1, PCK1, LINC00473, KCNG3, CHD5, RPL18AP7, MBOAT4, NXPH1, TRAJ33, TMEM200C, LINC02246, RHOXF1P3, DEFB1, GNG13, SIM1, KCNH2, PPP1R14C, GP2, MIR181A2HG, SLC6A17, GPR26, GABBR2, RN7SL269P, CCL11, GNG4, OPRD1, IGKV3D-15, RNU6-795P, CAMK2N2, SCN2A, SNAP25, RPS12P17, SYNGR3, FAM201A, RN7SL863P, SMCO2, SPRR3, S100B, ANKRD26P4, SLITRK2, RNU6-644P, CRABP1, KRT6A, ROBO2, ADAMTS19, LINC01554, HIST1H1A, SNORA33, CST2, HES5, LY6K, SCN3A, SNORA70E, SIX3, FDCSP, IGHV1-69, GLB1L2, RNY1P12, GSTM1, MIR7849, CBLN1, DLK1, TCN1, RN7SL304P, RMST, MKI67P1, CD300LG, IGLV4-60, LRP2, PATE4, PCSK2, MGAM2, KCNK3, SOX2, DPYSL5, HEPACAM2, PKNOX2		

RNA5SP225, RNA5SP134, RNA5SP514, RNA5SP338, MTRNR2L11, RNA5SP25, RNA5SP29, MTRNR2L12, RNA5SP74, ACE2, RNA5SP325, PCAT18, RNA5SP215, RNA5SP71, LINC00397, PRELID3BP3, LINC01234, FAR2P3, RNA5SP359, RNA5SP444, MAGEC1, TLE1P1, RNA5SP372, EYA4, TDRKH-AS1, SP8, GRTP1-AS1, RNA5SP192, HSD17B1P1, GLYATL1P4, RNU7-169P, GOLGA6B, FAM182B, MIR1301, MIR3942, KLF2P1, RNA5SP259, RNY3P16, RNA5SP490, SLC22A6, HM13-IT1, SCHLAP1, ARIH2OS, RN7SKP86, RNU6ATAC11P, ZPBP2, ABI1P1, C1orf105, TP53AIP1, RPS18P13, RNA5SP48, RNA5SP323, TNNC1, RNA5SP302, RNA5SP145, RNA5SP250, RNA5SP388, RNA5SP517, RNA5SP336, RNU6-759P, RNA5SP68, LINC00273, ACOT1, OR5C1, CKM, C22orf15, ZNF716, SMC2-AS1, AQP4, MYLPF, SMPD5, BIN2P1, RAPSN, WDR88, OR7E117P, GPR83, OR2B6, ACTBP9, ANXA10, NPAS1, RPL38P4, TMEM220-AS1, GRM5-AS1, IGHJ3P, BNIP3P24, LGALS8-AS1, RPL23AP90, LINC00483, LINC02363, TRMT112P3, NANOGP7, APOB, ANKRD20A18P, USP17L7, RNA5SP452, CLPSL2, PPFIA4, RAB5CP1, RN7SL749P, TMEM74B, YBX1P4, DLX4, NUTM2A, RNA5SP513, IGHD2-15, RNA5SP235, RP1, COL6A4P1, LIMD1-AS1, SLC45A2, CCDC74BP1, SCG3, RNU7-124P, RASL11A, CYP2T3P, LINC00689, SAMD11P1, IGHD3-3, LINC01634, RPGRIP1, TMPRSS7, MIR6769A, SPDYE22P, MEP1B, POTEF, SERHL, RN7SL180P, OSMR-AS1, HADHAP1, LINC01010, ANO1-AS2, DOC2A, CELA2B, TMEM92-AS1, GAPDHP2, SCRT1, ARHGAP40, LINC01544, GTF2IP13, ZNF209P, PLEKHD1, AQP4-AS1, RNA5SP301, FAM167B, TUBAL3, PSORS1C2, LINC01774, CELF3, BNC1, HSPB9, RN7SL733P, DPRXP5, ZC3H12B, TIPARP-AS1, KLHDC9, TMPRSS6, CRTC3-AS1, IKBKG, VDAC1P11, RPS23P6, EPX, PCDHB19P, MTRF1LP2, DMRT3, SAG, PTCD1, ARTN, EIF4A1P8, RN7SL32P, PGAM1P5, CFD, CCDC74B, HOXB-AS3, GJB7, POTEE, OR1K1, RPL5P34, RPS14P8, LINC01237, TGFB3-AS1, RAB6C, LIPE-AS1, TRAJ6, SNORD116-18, ASPRV1, ENO1P3, TMEM92, TPTE2P1, ASIC5, MRM1, ENPP7, PLEKHM1P1, SLCO4A1, CASK-AS1, RPL29P14, LRRC29, DNASE1L2, TNFRSF4, RAD51AP1P1, SRGAP3-AS2, SLC2A4, MAGIX, DLEU1-AS1, SMUG1-AS1, RN7SL612P, UCA1, STAG3L5P-PVRIG2P-PILRB, NWD1, SCN11A, TSSK3, LINC01133, ZNF709, PNPLA7, SLC2A1-AS1, MTND2P11, ADAT3, RNA5SP295, APBA3, CHRND, BTBD2, SLC16A8, LINC00484, PKD1L1, BHLHE22, RN7SKP292, RNU4-25P, MRPS18AP1, RN7SL164P, NME2P1, PCDHA12, RPS15AP10, CYP2F2P, RPSAP36, SMIM6, EDRF1-AS1, SCNN1B, OR5BA1P, DUSP14, TSPEAR-AS2, RN7SL812P, ANGPTL7, C8orf46, FAIM2,	Increased (*n* = 1198)	Sorokin[8]

PADI3, SCARNA15, ARSJ, RHOBTB2, ADAMTSL5, PABPC1P3, TMEM178A, RHEBL1, SH3RF2, C2orf42, SPX, RPL7P32, EPN3, PLIN5, LILRA1, LAMTOR5-AS1, HMGA1P4, GRIA4, NRL, ANKS3, IFFO1, SHANK2-AS3, ABHD11-AS1, CES3, ENPP7P4, IGHV2-70, BMPR1APS1, RN7SKP235, POLM, ZNF414, RN7SL263P, EPHA10, CPLX1, D2HGDH, GNB1L, LINC00222, GNMT, FAM66C, UPP2, TRIM7, TYRO3P, UNC5B-AS1, PLEKHH3, RPL7L1P3, ABCC3, WWOX-AS1, TET2-AS1, AP4M1, MEGF10, HNRNPA3P15, SRP14-AS1, CAPN12, CYP2J2, PROB1, TLE6, RN7SKP97, CCDC78, SIGLEC14, CBARP, PTCH2, PROC, PON3, ABCC6, RN7SL521P, THEM5, MXD3, PCED1A, CCDC152, MST1, NPM1P27, ITPK1-AS1, NATD1, DMKN, ATP6V0E2-AS1, NAGLU, LRRIQ1, C5orf66-AS2, P2RY11, ATP8B3, RNU6ATAC16P, CCDC144NL-AS1, ODCP, SLC26A6, FIGNL1, CDC20B, TNFAIP1, ETV5-AS1, SH2D3A, TUSC8, MINK1, HNRNPA1P68, XKRX, WASHC1, PKDREJ, IGLV3-10, SPATA20, RPL3P1, SERHL2, ADGRV1, MFSD10, C2orf72, RN7SKP275, PROSER2-AS1, SMC5-AS1, FCF1P8, ADAMTSL4-AS1, YBX2, ZNF70, KLC4, WNK4, METAP1D, FXN, FEN1, TAC4, SPRN, MIRLET7BHG, C1orf159, ZC3H12D, PIGL, STAB2, SCX, HOXB6, C20orf144, BAALC-AS1, MYH16, ZNF831, CPAMD8, SDR42E1, CLIC3, TAT, LINC01725, ATP2C2, ROR1, PLK2, HOXB5, ENTPD5, RNVU1-1, CAD, RTN4RL2, PODNL1, NYNRIN, PEX6, DHRS4L2, RNU7-181P, CRIP1, PHYHD1, KAT5, NUPR2, ZC3H4, FBXW12, EZH1, FBN3, HILPDA, LINC00672, CEP72, C9orf116, SRRM2-AS1, TOM1, SH2D2A, RNU1-139P, AMER1, ZNF350, FAM186B, BCAR3, CCDC146, NPIPP1, EXOC2, LRRN2, FPGS, SULT1A1, TNRC6C, PLEKHJ1, SLC26A1, UTP15, PHLDB3, GTF2IP20, LINC01569, CORO6, LINC00680, UNC5CL, STX2, RN7SL732P, UNC45A, TOP1MT, MAN2A2, DENND5B, NEU4, FSTL3, KRT81, PSMD9, EPHA3, RRAS2, ATP8A1, TMEM205, LYPD3, SLC28A3, ZDHHC4, SPA17, SYNGR1, ELL, ST20, OCIAD2, NUDT8, RASD1, SNORA71B, TIMM17B, ALDH3B1, NONOP2, TMEM216, MYL5, BMP2KL, LENG8-AS1, CTNS, RTEL1-TNFRSF6B, FBXO27, RAD51D, CHKB, ORAOV1, MYO1G, TM7SF2, SLIT2, GLDN, NSUN5, ZNF714, PRORSD1P, FCGR2C, RGL4, GPT, PSPN, RN7SL608P, ZNF646, CHERP, ABTB2, RPL18P10, PELI3, CHSY3, ATG2A, REEP6, RAB4A, LAS1L, UBAC1, CYR61, CROCC, C1QTNF3, PELI1, MINDY4, PCSK4, PKN3, TMEM94, MRRF, REXO1, GALE, ACSS2, EDN2, B9D2, HOXC4, ABHD11, CLDN12, FLRT1, HAVCR2, DVL1, SPATA24, RIPOR3, C15orf52, FNBP1P1, MAPK10, TMEM126B, GRB10, HIRIP3, CRYZ, PRSS16, C9orf16, WDR97, LZTS2, PMS1, INCENP, CYB561D1, EEF1D,		

ZNF826P, ACVR1B, RALGPS1, SYT2, NDE1, RN7SL431P, VAMP4, CARS2, C3orf62, ARHGEF17, ZNF439, TUBGCP6, KIF21A, RFX2, RN7SL660P, TMPRSS9, SP140L, TOLLIP, SNED1, PER2, GRIN1, KAZN, ZNF575, AMOTL2, NKX3-1, NIT1, RPUSD3, TMCC2, SLC25A37, OAZ3, DHX35, GATD1, MPV17, HAUS7, PUF60, C2orf54, ELOF1, ACOT9, UCK1, CDK5RAP3, KAT14, QSOX2, ERCC6L, BCL9, BAIAP2L2, EGFR, TANGO2, CHST3, LPIN1, BEST1, CISD3, ARHGAP29, ACOT2, EEF1A1P3, SCARNA22, TMEM198B, LARGE1, GOPC, MYO18A, SAMD11, MAPKAPK5-AS1, POU2F2, MORC4, NHSL2, KAT2B, TRO, SEPT1, MRPS31P5, MORN4, RAB3B, ACSF2, SLC35A4, ANKFY1, PNKD, RCAN1, FAM98A, CRBN, SETD1A, TAPT1-AS1, PLAU, FHL3, G6PD, ZNF517, RSAD1, ANKHD1, ZNF710-AS1, BOC, KCNK6, IGSF8, COMMD5, RN7SL141P, GPSM1, CBLB, ZNF839, MANBAL, TNPO2, IRF9, PSMG3, CCL5, BIN1, LRRC37B, FADS2, RASSF4, CUEDC1, ANO8, MYBBP1A, KIF21B, SPNS2, WNT7B, NUDT22, FRMD6-AS1, ZMYM3, ZNF44, RBL1, UPF1, PROX2, IDH3G, IKZF4, RPS7, C1orf116, TMC5, PRPF40B, PNPLA2, SIGLEC1, TBC1D22B, OBSCN, ZBTB7C, PIGQ, RRN3, RN7SL394P, TXNL4A, SLC25A1, RRP7BP, MARS, SYDE2, SLFNL1, ZDBF2, EIF3G, KIAA1324, LIMS2, MORN3, SAMD12, AP1G2, SNHG3, NEK7, TCOF1, HELLPAR, SRPK3, DPY19L1, SMARCC2, MICALL2, TXNDC15, GPR180, GGT6, WDFY4, TRIM2, AHCYL2, OTUD6B-AS1, GOLPH3L, IFFO2, SLC35E2B, TMEM132A, HLA-DQB2, PAQR7, LETM1, ZBTB7B, STX5, PTGIS, SNX17, IFT27, TOMM40L, FAH, CENPP, LINC00174, MRPL48, SPPL2B, RN7SKP180, COIL, CACNG4, CAMSAP3, MEF2C, SH3D21, TSHZ1, EPS8L1, CCDC61, KIAA0040, ASPSCR1, IL18BP, CCDC120, RBMS2, MTG1, TBC1D10C, NME4, CABLES1, SNX5, WNK2, N4BP2L1, INPP4A, NEAT1, ANKRD44-IT1, MUC1, ADGRF5, DONSON, TLDC1, ATG10, RASD2, NOXA1, EMC10, SHANK3, ADCK2, ZNF767P, RN7SL513P, SLC12A9, SLC4A5, RPL9, PHF21A, TTLL5, CDHR5, ACOX3, PRKCA-AS1, NFIX, C1QBP, CD37, C1QBPP2, LSM5, ADGRA2, FRMPD3, RABIF, CPT1B, FAM53C, TNFSF15, CCNDBP1, B3GNT5, ZNRF1, PLD2, SLX4, TIMM21, FUK, SETDB1, RNF146, GABARAPL1, ATXN10, SLC12A6, TLN2, REPIN1, AKT1S1, TMC4, SYTL4, DNAJB5, ZNF581, BCAP31, SF3A2, HKR1, CBWD2, PATJ, NADSYN1, RAPGEF5, SIPA1, FYTTD1, IL6R, RRP7A, EME2, LONRF1, FBXL19, RNU1-11P, RPAIN, RAB13, SELENOO, ERO1B, STX1B, RAB11FIP1, DEDD2, KRT8, ZC3H12C, DOCK6, ATAD3B, ARFRP1, GSTK1, FZR1, RAPH1, TRMT2A, FBXO44, BTF3, LINC-PINT, RRP8, SGPP1, FECH, TLE2, RPS20P22, SKAP2, MTA1, FBXO25, NPAS2, EHMT2, PANK2,		

CLDN3, ALDH1A3, FBXO46, ARHGAP5, TMEM150A, SHC1, ACIN1, MORF4L1, LMTK3, LTBP3, TXNRD2, TRMT2B, DNAJC17, RAD51, ZNF737, TMEM120B, TOB1, YARS2, NCOR2, TYW5, DUSP18, CAPRIN2, B3GNT7, LINC00969, MAMDC4, COL27A1, BOK, SYNE3, NDUFV3, MICAL3, GLTP, CHD6, FANK1, TRIT1, HSPB7, ZNF793, DEDD, FUZ, ANKRD49, RNF24, KIAA1191, SLC25A45, SCAF4, TNFAIP3, DVL3, BORCS8, NANOS1, SLC43A2, USP18, VIPR1, PIGU, CAMTA2, DDX27, SSPN, SERPINB1, BAHD1, SHROOM1, NECTIN4, SELENOS, DDR1, RAB35, MAP4K2, NAT10, NUPR1, PGGHG, IPO13, SLC35C2, SAFB2, GTPBP6, RNPS1, ADAP2, FTH1, NRSN2, TRMT10C, EME1, PCDH12, UBE2G1, PSMB6, SHANK2-AS1, SCMH1, SLC22A23, WDR91, ABCF1, ARHGEF11, NR2C2, ADAMTSL4, RPL17, SSBP2, ST5, IL17RC, PAF1, BRD2, MRPL30, S1PR2, CNTN2, SMPD1, LLPH, MAP3K6, UBXN6, APBB3, SARM1, PPP1R3F, NUP160, FBXO32, GTF2E1, TNRC18, TK1, TJAP1, GUSBP1, ARID1A, B4GALT3, E4F1, PLA2G6, RBSN, FBRSL1, ACTR1B, CRIP2, MFN2, MCRIP2, SLFNL1-AS1, ZNF706, CEP120, TNS2, ODF3B, MAPRE3, TAPT1, BAX, RNF19A, SLC2A11, NBEAL2, ABCA7, BDKRB2, HARS, ETF1, UNC13D, BICRA, DNAJC18, THAP6, RPS3A, MTMR10, NAB2, ADGRL1, AVL9, FUT2, DLG4, CLK2, ZNF33B, KRT7, THBS4, PHAX, USP43, PIM3, RAB11FIP5, PKP3, LASP1, LRCH1, ECI1, CYB561, TMEM64, MAP3K12, POFUT1, CENPBD1P1, KAT2A, NDUFA3, ZNF619, COLGALT1, FBXO41, MAPK8IP3, SGTA, SLC15A3, SDHC, LUC7L3, GSDMD, FAM160B2, SPRY1, BNIPL, FAM219A, LEMD3, MYO19, H6PD, RPS17, NFYA, TCF3, TSPOAP1, GUK1, EXD3, SMIM5, MCM3AP, BLOC1S2, SMG1P3, DIAPH1, MTM1, TRIQK, SHKBP1, SLCO2B1, TMEM63C, NOP53, MYDGF, NDUFA12, MTMR1, ZMYND8, SMTN, KLHL24, RAB3D, KIF1C, PLEKHG3, RETSAT, RAB40C, PITPNM3, GPKOW, SLC41A1, FBXO18, MAFK, ERGIC3, FRYL, GJC1, STXBP1, MRPL24, LTBR, PIP5K1C, MADD, FLCN, ZNF431, RBM14, SGSM2, ZNF7, MBD6, HMBOX1, CA5B, SERF2, AP3D1, MEF2A, PELP1, CAMKK1, FASTK, TLE1, GNL3L, CABIN1, TMEM219, GALNT2, RAB11FIP1P1, PTCD2, ZNF276, GNA12, PROM2, MNT, CERS5, CIRBP, CLINT1, HIF1AN, CNOT3, ALAD, ZNF207, HEATR6, TBC1D22A, PPP1R12B, UTP6, BRD1, IGSF9, PPRC1, TNS3, SNX1, RBFOX2, HMG20B, AARS2, CPSF1, ZMIZ2, RGS12, MAT2A, ERICH1, FAM13A-AS1, GOLGA2, SLC35B2, AP5Z1, CCDC125, CLPTM1, CTPS1, GIGYF1, YPEL2, ENO1, VARS, CDC42EP1, TWNK, MOV10, SLC3A2, PIAS3, MAP3K11, RHOT2, CDK13, ALDOA, GLI4, CREBBP, PSMD1, FAM189B, VMP1, ANKRD52, PEG13, ZNF621, ZNF496, RGL2, ZNF281, GGA3, KMT2C,		

TRIM47, FLOT1, MBD1, SMURF1, INTS11, CREB3L4, KANSL3, ZBTB7A, ASS1, CEP104, ABT1, LTBP1, GRHL2, VLDLR, GYS1, LMAN2, TPRN, TMEM259, ZNF814, DDX56, CLUH, CCNG2, ACAP3, WWC1, EHF, ARHGAP27, ARSG, B4GALNT4, DHX34, EIF2B4, TCIRG1, RAPGEF3, TECPR2, SPEN, SLC16A2, RBCK1, ZC3H7B, LINC00963, SLC2A10, ZNF740, SAR1B, ZNF514, TRIM62, INTS1, ZFYVE26, ZNF552, CYB561A3, TPCN1, DCAF11, COPA, CTSD, MED25, KMT2D, NUMB, FBXW4, SRGAP3, BCL9L, GAS2L1, TRIM25, VPS37B, RAB1B, SZT2, PTRH2, TMEM214, ZNFX1, JUN, R3HDM4, PRRC2A, PARP10, FNBP4, POLR3E, REV1, MALAT1, EHMT1, CARHSP1, FUS, DAZAP1, TMC6, LIG3		
AAK1, ABAT, ABCA2, ABCB1, ABI3BP, ACAP1, ACSL5, ADHFE1, ADPGK, ADRB2, AFAP1L2, AMICA1, ANK2, ANKRD44, AOAH, APOBEC3D, APOBEC3H, APOD, APOL3, AR, ARHGAP15, ARHGAP9, ARIH2, ARRDC5, ATAD1, BATF2, BATF3, BIRC3, BLR1, BMP2K, BTK, BTN3A1, C12orf65, C14orf139, C17orf108, C2, C20orf100, C3, C3orf71, C4orf21, C4orf23, C5orf20, C5orf39, CASP5, CCDC69, CCL19, CCL21, CCL5, CCM2, CCNH, CCNL1, CCR10, CCR2, CCR4, CCR6, CCR7, CD160, CD1C, CD1E, CD2, CD247, CD27, CD274, CD37, CD38, CD3D, CD3E, CD3G, CD40LG, CD48, CD52, CD6, CD69, CD72, CD79A, CD79B, CD80, CD8A, CD96, CD97, CLEC10A, CLEC2B, CLEC2D, CLEC4C, CNOT6L, CPXM2, CTLA4, CXCL12, CXCL13, CXCR3, CXCR4, CXCR5, DAAM2, DAZAP2, DENND3, DNAH1, DOC2B, DOCK10, DPEP2, DPPA4, EAF2, EBI3, EDARADD, EIF4A3, EIF4E3, EIF4ENIF1, ELOVL5, ENPP2, EPSTI1, EVI2B, FAM115C, FAM125B, FAM46C, FAM65B, FCRL1, FCRL3, FCRLA, FECH, FERMT3, FGL2, FNBP1, FOXA1, FYN, GALM, GFI1, GIMAP2, GIMAP4, GIMAP5, GIMAP6, GIMAP7, GIMAP8, GIT2, GNB1L, GPR171, GPR174, GPR55, GRAP, GZMA, GZMB, GZMH, GZMK, HAVCR1, HCLS1, HCST, HEMK1, HLA-DOB, HLA-DPB1, HMHA1, HPS1, HSPC157, ICAM3, ICOS, IDO1, IFT57, IGF1, IGLL1, IL10RA, IL11RA, IL12RB1, IL21R, IL2RB, IL2RG, IL7R, INDO, INPP5D, IRF4, IRF8, ISG20, ITGAL, ITGB2, ITK, ITPRIPL1, IVD, JMJD1C, KCNA3, KCNJ10, KIF21B, KLRB1, KLRC3, KLRG1, LARS2, LAX1, LCK, LEPR, LILRA6, LIMD2, LOC388692, LOC440957, LPXN, LTA, LTB, LY75, LY9, LYZ, MAP3K14, MAP4K1, MAPKAPK3, MATK, MBLAC1, MFNG, MGC40069, MGST2, MIR155HG, MIST, MLH1, MLL5, MLPH, MN1, MTUS1, MUSTN1, NCKAP1L, NCOA2, NCR1, NICN1, NKG7, NLRC3, NLRP1, NOD3, NR5A2, NTNG2, NUAK2, OSCAR, P2RX1, P2RY10, P2RY14, P2RY8, PARVG, PDGFD, PECAM1, PHF17, PIM1, PIM2, PIP4K2A, PLA1A, PLCG2, PLCL1, PLCL2,	Decreased (*n* = 330)	Perez[9]

PLEK, PLEKHO2, POLG, PPAPDC1B, PPARG, PPP1R16B, PRF1, PRKCA, PRKCB1, PRKCH, PRKCQ, PROCA1, PRR22, PSCD4, PTCRA, PTGDR, PTGDS, PTGER4, PTPN22, PTPRC, PTPRCAP, PVRIG, PYHIN1, QRICH1, RAB37, RALGAPA1, RASGRP2, RBM17, RCSD1, RNF19A, SAMD3, SAMD9L, SARM1, SELL, SELP, SEMA4A, 38961, SERHL2, SH2D1A, SH2D2A, SH3TC1, SHD, SLA2, SLAMF6, SLC10A5, SLC14A1, SLIT2, SP140, SPN, SPOCK2, SS18L1, ST3GAL5, STAP1, STARD5, STAT6, TAGAP, TCF7, TCL1A, TFF3, TGFBR2, TIAM1, TIFA, TIGIT, TLR10, TLR9, TM6SF1, TMEM156, TNFAIP8, TNFRSF13C, TNFRSF1B, TNFRSF9, TP53INP1, TSPAN32, TSPAN7, TXNIP, UBD, UBE2G1, UCKL1, USP50, UTRN, VAMP1, VCAM1, WAS, WDR33, XBP1, ZBTB32, ZFP30, ZNF425, ZNF470, ZNF655, ZNF683, ZNF799, ZNF831		
ADAM17, ADAM22, AGRN, AIF1L, ALS2CR12, ANK3, ANKRD36B, ANKRD38, ANKRD7, APCDD1L, ATF6B, ATL3, ATP1A1, ATRN, BBOX1, BCAR1, BCAS2, C18orf45, C1orf133, C5orf46, CAB39L, CAPRIN1, CBX1, CCDC147, CCDC90A, CCNL2, CDH2, CHCHD3, CHRNA3, CPNE2, CRY1, CSDE1, CSPG4, CYP2A13, CYP2D7P1, DCLK2, DCPS, DDX27, DHX29, DHX40, DKFZp761E198, DSC2, EFS, EFTUD2, ENHO, ERCC8, FAM187B, FAM25C, FAM25G, FAM83B, FGF1, GABRP, GLI3, GRHL1, HAO1, HIF1A, HIST1H2AC, HIST1H3D, HOMER1, IHPK3, IL17RC, INHBA, INS, ITGA11, JUP, KCNC4, KIAA0427, KIF26B, KLK5, LAMB3, LAMC2, LCLAT1, LDLRAD3, LIMS1, LOC285359, LOC441376, LOC642636, LOC730243, LSG1, MAGEC3, MAP9, MAPK8, MBIP, MCM8, MLF1, MMACHC, MRPL49, MSH2, MTA3, MTSS1L, NACC1, NOC4L, N-PAC, OGFRL1, OR8U1, P2RX2, PALM2, PCSK1N, PCSK6, PHF6, PIAS2, PIR, PKP3, PLAUR, PPP1CB, PSMB7, PSMC3IP, PSMD14, PTPN20A, PTPRF, PTPRK, PUS3, QSOX1, RAB12, RARG, RHBDL2, RHCE, RNF186, RPL13, RTN4, SAA2, SCUBE3, SDCBP2, SEC22B, SEMA4B, SERPINB5, SERPINH1, SHB, SIP1, SLC34A2, SLC5A9, SLC6A2, SMYD5, SNORD77, SNX12, SORBS1, SS18, SYCE1L, TARBP2, TBC1D24, TCEAL1, TDRD3, TGM5, TMEM127, TMEM167A, TMTC3, TOE1, TRIM16, TWF1, ULK1, VGLL1, VWA1, WNK2, WNT11, WWOX	Increased (n = 155)	Perez[9]

Supplementary Table 12. Pathways associated with differentially expressed genes

**Pathway**	**Reference**
PI3K pathway<br>EGF receptor signaling pathway	Gámez-Pozo

Apoptosis signaling pathway<br>p53 pathway<br>Hypoxia response via HIF activation	[2]
ATM_Pathway<br>ATM_Pathway_Cell_Survival<br>Base_Excision_Repair_Pathway<br>biocarta_activation_of_camp_dependent_protein_kinase_pka_Main_Pathway<br>biocarta_activation_of_csk_by_camp_dependent_protein_kinase_inhibits_signaling_through_the_t_cell_receptor_Main_Pathway<br>biocarta_akap95_role_in_mitosis_and_chromosome_dynamics_Main_Pathway<br>biocarta_akap95_role_in_mitosis_and_chromosome_dynamics_Pathway_.mitotic_chromosome_condensation.<br>biocarta_akap95_role_in_mitosis_and_chromosome_dynamics_Pathway_.mitotic_chromosome_decondensation.<br>biocarta_akt_signaling_Main_Pathway<br>biocarta_angiotensin_ii_mediated_activation_of_jnk_pathway_via_pyk2_dependent_signaling_Main_Pathway<br>biocarta_attenuation_of_gpcr_signaling_Main_Pathway<br>biocarta_chrebp_regulation_by_carbohydrates_and_camp_Main_Pathway<br>biocarta_cystic_fibrosis_transmembrane_conductance_regulator_cftr_and_beta_2_adrenergic_receptor_b2ar_Main_Pathway<br>biocarta_how_progesterone_initiates_the_oocyte_maturation_Main_Pathway<br>biocarta_how_progesterone_initiates_the_oocyte_maturation_Pathway_.oocyte_maturation.<br>biocarta_keratinocyte_differentiation_Main_Pathway<br>biocarta_mapkinase_signaling_Main_Pathway<br>biocarta_mcalpain_and_friends_in_cell_motility_Main_Pathway<br>biocarta_mechanisms_of_transcriptional_repression_by_dna_methylation_Main_Pathway<br>biocarta_mechanisms_of_transcriptional_repression_by_dna_methylation_Pathway_.histone_deacetylation.<br>biocarta_multi_step_regulation_of_transcription_by_pitx2_Main_Pathway<br>biocarta_phospholipids_as_signalling_intermediaries_Main_Pathway<br>biocarta_protein_kinase_a_at_the_centrosome_Main_Pathway<br>biocarta_role_of_egf_receptor_transactivation_by_gpcrs_in_cardiac_hypertrophy_Main_Pathway<br>biocarta_role_of_erbb2_in_signal_transduction_and_oncology_Main_Pathway<br>biocarta_role_of_nicotinic_acetylcholine_receptors_in_the_regulation_of_apoptosis_Main_Pathway<br>biocarta_signaling_pathway_from_g_protein_families_Main_Pathway<br>biocarta_sprouty_regulation_of_tyrosine_kinase_signals_Main_Pathway<br>biocarta_stathmin_and_breast_cancer_resistance_to_antimicrotubule_agents_Main_Pathway<br>biocarta_tgf_beta_signaling_Main_Pathway<br>biocarta_transcription_factor_creb_and_its_extracellular_signals_Main_Pathway<br>biocarta_trefoil_factors_initiate_mucosal_healing_Main_Pathway	Sorokin[8]

Calcium2._Signaling<br>cAMP_Pathway<br>cAMP_Pathway_Axonal_Growth<br>cAMP_Pathway_Cell_Proliferation<br>cAMP_Pathway_Degradation_of_Cell_Cycle_Regulators<br>cAMP_Pathway_eNOS_Signaling_Cardiovascular_Homeostasis<br>cAMP_Pathway_Gene_Expression_via_NFKB2_CREBBP_ELK1<br>cAMP_Pathway_Glycogen_Synthesis<br>cAMP_Pathway_Lipolysis<br>cAMP_Pathway_Oncogenesis<br>cAMP_Pathway_Protein_Retention<br>cAMP_Pathway_Regulation_of_Cytoskeleton<br>Chemotaxis_Driven_by_CCL2<br>Chemotaxis_Driven_by_IL.8_and_LTB4<br>EGF_Pathway<br>EGF_Pathway_Gene_Expression_via_FOS_NFKB2_MYC_STAT1_ELK1_STAT3_JUN<br>ErbB_Family_Pathway<br>Estrogen_Pathway<br>Estrogen_Pathway_Gene_Expression_via_FOS_JUN_ELK1_SP1_POLR2B_CREB3_NFKB2<br>FGFR_Signaling<br>FLT3_Signaling_Pathway<br>FLT3_Signaling_Pathway_Transcription_via_ELK3_MAPK12_CREB3_STAT2<br>gluconeogenesis<br>Growth_Hormone_Signaling_Pathway<br>GSK3_Signaling_Pathway_Glycogen_Synthesis<br>GSK3_Signaling_Pathway_Protein_Synthesis<br>HGF_Pathway<br>HIF1Alpha_Pathway<br>HIF1Alpha_Pathway_Gene_Expression_via_JUN_CREB3<br>HMGB1_signaling_through_RAGE<br>IGF1R_Signaling_Pathway_Cell_Survival<br>IGF1R_Signaling_Pathway_Protein_Synthesis<br>ILK_Signaling_Pathway_Loss_of_Occludin_Barrier_Dysfunction<br>ILK_Signaling_Pathway_MMP2_MMP9_Gene_Expression_Tissue_Invasion_via_FOS<br>ILK_Signaling_Pathway_Tissue_Morphogenesis<br>KEGG_Adherens_junction_Main_Pathway<br>KEGG_Adipocytokine_signaling_Main_Pathway	

KEGG_Adrenergic_signaling_in_cardiomyocytes_Main_Pathway<br>KEGG_Alanine_aspartate_and_glutamate_metabolism_Main_Pathway<br>KEGG_Apoptosis_Main_Pathway<br>KEGG_Base_excision_repair_Main_Pathway<br>KEGG_beta_Alanine_metabolism_Main_Pathway<br>KEGG_cAMP_signaling_Main_Pathway<br>KEGG_Central_carbon_metabolism_in_cancer_Main_Pathway<br>KEGG_Chagas_disease_American_trypanosomiasis__Main_Pathway<br>KEGG_Chemical_carcinogenesis_Main_Pathway<br>KEGG_Choline_metabolism_in_cancer_Main_Pathway<br>KEGG_Drug_metabolism_cytochrome_P450_Main_Pathway<br>KEGG_Drug_metabolism_other_enzymes_Main_Pathway<br>KEGG_Epstein_Barr_virus_infection_Main_Pathway<br>KEGG_ErbB_signaling_Main_Pathway<br>KEGG_Estrogen_signaling_Main_Pathway<br>KEGG_Focal_adhesion_Main_Pathway<br>KEGG_GABAergic_synapse_Main_Pathway<br>KEGG_Glioma_Main_Pathway<br>KEGG_Hepatitis_B_Main_Pathway<br>KEGG_Hepatitis_C_Main_Pathway<br>KEGG_Herpes_simplex_infection_Main_Pathway<br>KEGG_Leukocyte_transendothelial_migration_Main_Pathway<br>KEGG_Lysosome_Main_Pathway<br>KEGG_MAPK_signaling_Main_Pathway<br>KEGG_MicroRNAs_in_cancer_Main_Pathway<br>KEGG_Non_alcoholic_fatty_liver_disease_NAFLD__Main_Pathway<br>KEGG_Non_homologous_end_joining_Main_Pathway<br>KEGG_Pancreatic_cancer_Main_Pathway<br>KEGG_Pantothenate_and_CoA_biosynthesis_Main_Pathway<br>KEGG_Pentose_and_glucuronate_interconversions_Main_Pathway<br>KEGG_Prolactin_signaling_Main_Pathway<br>KEGG_Prostate_cancer_Main_Pathway<br>KEGG_Protein_digestion_and_absorption_Main_Pathway<br>KEGG_Protein_export_Main_Pathway<br>KEGG_Protein_processing_in_endoplasmic_reticulum_Main_Pathway<br>KEGG_Pyrimidine_metabolism_Main_Pathway<br>KEGG_Renin_angiotensin_system_Main_Pathway	

KEGG_Retinol_metabolism_Main_Pathway<br>KEGG_Selenocompound_metabolism_Main_Pathway<br>KEGG_Sphingolipid_metabolism_Main_Pathway<br>KEGG_Taurine_and_hypotaurine_metabolism_Main_Pathway<br>KEGG_TNF_signaling_Main_Pathway<br>KEGG_Ubiquitin_mediated_proteolysis_Main_Pathway<br>MAPK_Family_Pathway_Chromatin_Remodelling<br>MAPK_Family_Pathway_Gene_Expression_via_ATF2_JUN_ELK1_NFKB2_CREB3<br>MAPK_Signaling_Pathway_Gene_Expression_Apoptosis_Inflammation_Tumorigenesis_via_MYC_HSF1_STAT2<br>MAPK_Signaling_Pathway_Gene_Expression_Cell_Proliferation_Cell_Survival_Tumorigenesis_Differentiation_Development_via_PXN_CREB3_RPS6KA5_RPS6KA6<br>melatonin_degradation_I<br>mTOR_Pathway_Translation_Elongation<br>NCI_amb2_Integrin_signaling_Main_Pathway<br>NCI_BCR_signaling_Pathway_.cytokine_secretion.<br>NCI_CD40_CD40L_signaling_Main_Pathway<br>NCI_CDC42_signaling_events_Main_Pathway<br>NCI_Class_I_PI3K_signaling_events_mediated_by_Akt_Pathway_.negative_regulation_of_cell_cycle.<br>NCI_DNA_PK_pathway_in_nonhomologous_end_joining_Main_Pathway<br>NCI_DNA_PK_pathway_in_nonhomologous_end_joining_Pathway_.double_strand_break_repair_via_nonhomologous_end_joining.<br>NCI_DNA_PK_pathway_in_nonhomologous_end_joining_Pathway_.V_D_J_recombination.<br>NCI_ErbB2_ErbB3_signaling_events_Pathway_.myelination.<br>NCI_FAS_CD95_signaling_Main_Pathway<br>NCI_Hedgehog_signaling_events_mediated_by_Gli_proteins_Main_Pathway<br>NCI_IL12_signaling_mediated_by_STAT4_Main_Pathway<br>NCI_LKB1_signaling_events_Main_Pathway<br>NCI_LKB1_signaling_events_Pathway_.Pathway_positive_regulation_of_CREB_transcription_factor_activity_via_CRTC2.<br>NCI_LPA_receptor_mediated_events_Main_Pathway<br>NCI_Notch_signaling_Main_Pathway<br>NCI_p38_signaling_mediated_by_MAPKAP_kinases_Main_Pathway<br>NCI_PDGFR_beta_signaling_Main_Pathway<br>NCI_Posttranslational_regulation_of_adherens_junction_stability_and_dissassembly_Main_Pathway<br>NCI_Posttranslational_regulation_of_adherens_junction_stability_and_dissassembly_Pathway_.axonogenesis.<br>NCI_Presenilin_action_in_Notch_and_Wnt_signaling_Pathway_.apoptosis.	

NCI_RAC1_signaling_Main_Pathway<br>NCI_Regulation_of_CDC42_activity_Main_Pathway<br>NCI_Regulation_of_nuclear_SMAD2_3_signaling_Pathway_.muscle_cell_differentiation.<br>NCI_SHP2_signaling_Main_Pathway<br>NCI_Signaling_events_mediated_by_focal_adhesion_kinase_Main_Pathway<br>NCI_Signaling_events_mediated_by_HDAC_Class_I_Pathway_.histone_deacetylation.<br>NCI_Signaling_events_mediated_by_Hepatocyte_Growth_Factor_Receptor_c_Met__Main_Pathway<br>NCI_Signaling_events_mediated_by_TCPTP_Main_Pathway<br>NCI_Signaling_mediated_by_p38_alpha_and_p38_beta_Main_Pathway<br>NCI_Stabilization_and_expansion_of_the_E_cadherin_adherens_junction_Pathway_.actin_cable_formation.<br>NCI_Validated_transcriptional_targets_of_TAp63_isoforms_Main_Pathway<br>NCI_Validated_transcriptional_targets_of_TAp63_isoforms_Pathway_.Metastasis.<br>NCI_Validated_transcriptional_targets_of_TAp63_isoforms_Pathway_.Pathway_degradation_of_TP63.<br>NGF_Pathway<br>Nitric_Oxide_Pathway_in_Skeletal_Muscle<br>noradrenaline_and_adrenaline_degradation<br>p53_Signaling_Pathway<br>p53_Signaling_Pathway_Apoptosis<br>p53_Signaling_Pathway_Breast_Cancer<br>p53_Signaling_Pathway_Cancer<br>p53_Signaling_Pathway_Cell_Cycle_Arrest<br>p53_Signaling_Pathway_Cell_Growth_Accumulation<br>p53_Signaling_Pathway_DNA_Repair<br>p53_Signaling_Pathway_Exosome_Mediated_Secretion<br>p53_Signaling_Pathway_Gene_Expression_Anti.Apoptosis_via_TP53<br>p53_Signaling_Pathway_Gene_Expression_Cell_Cycle_and_Cell_Growth_via_TP53<br>p53_Signaling_Pathway_Gene_Expression_Cell_Fate_and_Development_via_TP53<br>p53_Signaling_Pathway_Gene_Expression_Cell_Signaling_via_TP53<br>p53_Signaling_Pathway_Gene_Expression_DNA_Replication_and_Repair_via_TP53<br>p53_Signaling_Pathway_Gene_Expression_ECM_and_Adhesion_via_TP53<br>p53_Signaling_Pathway_Gene_Expression_Infection_and_Immune_Response_via_TP53<br>p53_Signaling_Pathway_Inhibition_of_Angiogenesis_and_Metastasis<br>p53_Signaling_Pathway_Normal_Cell_Cycle_Progression<br>PPAR_Pathway<br>PTEN_Pathway_Adhesion_or_Migration<br>RANK_Signaling_in_Osteoclasts_Pathway<br>RANK_Signaling_in_Osteoclasts_Pathway_Expression_of_Osteoclastic_Genes_via_JUN_NFAT5_NFKB2_MIT	

F_FOS<br>Ras_Pathway_Apoptosis<br>reactome_Activation_of_DNA_fragmentation_factor_Main_Pathway<br>reactome_Adherens_junctions_interactions_Main_Pathway<br>reactome_Amyloids_Main_Pathway<br>reactome_Antigen_Presentation_Folding_assembly_and_peptide_loading_of_class_I_MHC_Main_Pathway<br>reactome_APC_Cdc20_mediated_degradation_of_Nek2A_Main_Pathway<br>reactome_Budding_and_maturation_of_HIV_virion_Main_Pathway<br>reactome_Circadian_Clock_Main_Pathway<br>reactome_Class_C_3_Metabotropic_glutamate_pheromone_receptors__Main_Pathway<br>reactome_Collagen_degradation_Main_Pathway<br>reactome_Condensation_of_Prophase_Chromosomes_Main_Pathway<br>reactome_Cytosolic_tRNA_aminoacylation_Main_Pathway<br>reactome_DARPP_32_events_Main_Pathway<br>reactome_Dermatan_sulfate_biosynthesis_Main_Pathway<br>reactome_DNA_Damage_Telomere_Stress_Induced_Senescence_Main_Pathway<br>reactome_Endosomal_Sorting_Complex_Required_For_Transport_ESCRT__Main_Pathway<br>reactome_EPHB_mediated_forward_signaling_Main_Pathway<br>reactome_Ephrin_signaling_Main_Pathway<br>reactome_Fatty_acids_Main_Pathway<br>reactome_FCERI_mediated_Ca_2_mobilization_Main_Pathway<br>reactome_Formation_of_ATP_by_chemiosmotic_coupling_Main_Pathway<br>reactome_Glycosphingolipid_metabolism_Main_Pathway<br>reactome_ISG15_antiviral_mechanism_Main_Pathway<br>reactome_Meiotic_recombination_Main_Pathway<br>reactome_Meiotic_synapsis_Main_Pathway<br>reactome_Metabolism_of_Angiotensinogen_to_Angiotensins_Main_Pathway<br>reactome_Mitochondrial_tRNA_aminoacylation_Main_Pathway<br>reactome_Mitotic_Prometaphase_Main_Pathway<br>reactome_MyD88_Mal_cascade_initiated_on_plasma_membrane_Main_Pathway<br>reactome_Negative_regulators_of_RIG_I_MDA5_signaling_Main_Pathway<br>reactome_NoRC_negatively_regulates_rRNA_expression_Main_Pathway<br>reactome_Oxidative_Stress_Induced_Senescence_Main_Pathway<br>reactome_Packaging_Of_Telomere_Ends_Main_Pathway<br>reactome_PKA_activation_in_glucagon_signalling_Main_Pathway<br>reactome_Post_chaperonin_tubulin_folding_Main_Pathway<br>reactome_PRC2_methylates_histones_and_DNA_Main_Pathway	

reactome_Recruitment_of_NuMA_to_mitotic_centrosomes_Main_Pathway<br>reactome_Regulation_of_cholesterol_biosynthesis_by_SREBP_SREBF__Main_Pathway<br>reactome_repression_of_WNT_target_genes_Main_Pathway<br>reactome_Resolution_of_Sister_Chromatid_Cohesion_Main_Pathway<br>reactome_RIG_I_MDA5_mediated_induction_of_IFN_alpha_beta_pathways_Main_Pathway<br>reactome_RNA_Polymerase_I_Chain_Elongation_Main_Pathway<br>reactome_RNA_Polymerase_I_Promoter_Opening_Main_Pathway<br>reactome_RNA_Polymerase_II_Pre_transcription_Events_Main_Pathway<br>reactome_Sema4D_induced_cell_migration_and_growth_cone_collapse_Main_Pathway<br>reactome_Separation_of_Sister_Chromatids_Main_Pathway<br>reactome_SHC1_events_in_ERBB2_signaling_Main_Pathway<br>reactome_Signaling_by_constitutively_active_EGFR_Main_Pathway<br>reactome_Signaling_by_Hippo_Main_Pathway<br>reactome_Signaling_by_NODAL_Main_Pathway<br>reactome_The_activation_of_arylsulfatases_Main_Pathway<br>reactome_The_NLRP3_inflammasome_Main_Pathway<br>reactome_Trafficking_of_AMPA_receptors_Main_Pathway<br>reactome_Transport_of_organic_anions_Main_Pathway<br>reactome_Vasopressin_regulates_renal_water_homeostasis_via_Aquaporins_Main_Pathway<br>reactome_XAV939_inhibits_tankyrase_stabilizing_AXIN_Main_Pathway<br>reactome_Xenobiotics_Main_Pathway<br>Regulation_of_Cytoskeleton_Remodeling_by_Activin_A<br>Regulation_of_Cytoskeleton_Remodeling_by_TGF_and_WNT<br>Regulation_of_Repulsive_Axonal_Pathfinding<br>Role_of_NTN1_in_Axon_Pathfinding<br>superpathway_of_pyrimidine_deoxyribonucleotides_ide_novoi_biosynthesis<br>superpathway_of_pyrimidine_ribonucleotides_ide_novoi_biosynthesis<br>TGF.Beta_Pathway_Transciption_Arrested_Growth_Apoptosis<br>TGF.Beta_Pathway_Transciption_Cell_Growth_and_Mobility_and_Angiogenesis<br>tRNA_charging<br>UTP_and_CTP_ide_novoi_biosynthesis<br>WNT_Pathway_Cell_Survival<br>WNT_Pathway_NFAT_Pathway<br>X3.phosphoinositide_biosynthesis	
Immune-system related pathways<br>Proliferation-associated pathways	Triulzi [3]
Wnt signaling	Khour

Notch signaling	y[1]*
Angiogenesis<br>Notch signaling	Guarn eri[7]*

*Gene ontology analysis using the online available software PANTHER.

Supplementary Table 13. MicroRNA (miRNA) differentially expressed in breast cancer tissues of cases compared to controls

miRNA	Direction change in gene expression in resistant tumors	Reference
hsa-miR-361-5p, hsa-miR-26a-5p, hsa-miR-365a-3p, hsa-miR-155-5p, hsa-miR-205-5p, hsa-miR-150-5p, hsa-miR-106b-5p	Increased	Du[10]
hsa-miR-4734, hsa-miR-424-3p	Decreased	Du[10]
miR-210, miR-31-3p, miR-449a, miR-449b-5p	Increased	Ohzawa[11]
miR-106b-3p, miR-1180, miR-1238-5p, miR-142-5p, miR-150-5p, miR-181c-5p, miR-182-5p, miR-200a-5p, miR-218-5p, miR-3609, miR-362-5p, miR-3620-3p, miR-4418, miR-4506, miR-4657, miR-505-3p, miR-505-5p	Decreased	Ohzawa[11]

miRNA: microRNA.

Supplementary Table 14. Genes that have been reported to show epigenetic and/or genetic alterations in breast cancer tissues or blood of cases compared to controls and that have been predicted to be targeted by miRNA identified as differentially expressed in breast cancer tissues of cases compared to controls

	Genes that are predicted to be targeted by miRNA identified as differentially expressed in breast cancer tisues of cases compared to controls in the study of Ohzawa and collaborators (12) and Du and collaborators (10) that have been reported to show epigenetic and/or genetic alterations	Reference
Differentially methylated genes	ZNF598, KCNH7, ADAMTS2, SIX2, DOCK1	Furrer [2022]

Differentially expressed gene (at mRNA levels) in at least two studies	ABT1, ANKRD52, ARL3, ATP8A1, BCL9, BLOC1S2, BRD1, C1QTNF3, CARHSP1, CASP1, CDK15, CLEC14A, COMMD10, COMMD5, COMMD6, DAZAP1, DEDD, DPY19L1, DUSP14, EMC10, FEN1, FRK, GALNT2, GGA3, HMCN1, HOXC4, IKZF3, IPMK, IRAK3, IRF9, KIF1C, LASP1, MBD6, MORN2, MYO18A, PAQR7, PLEKHG3, POFUT1, PROM2, PRSS16, RAB4A, RAD51, RNF146, RNF24, SGPP1, SHROOM1, SIKE1, SIX1, SLC25A37, SLC2A10, SLC35B2, SLC41A1, STT3A, SYTL4, TOM1L1, VLDLR, VMP1, WNT7B, ZNF281, ZNF439, ZNF740	Sorokin[8], Merry[4]
	ADAM22, CTLA4, FECH, FGL2, KIF21B, LAMC2, NR5A2, WNK2, ZNF831	Sorokin[8], Perez[9]
	ACSL5, ATRN, FOXA1, MLPH, NACC1, PCSK6, PIM2, PLAUR, RHBDL2, SORBS1, TCEAL1, XBP1	Merry[4], Perez[9]
	ACBD3, AMMECR1, ARHGDIA, ATP6V0B, CD48, ENAH, FOSL2, GNPAT, KDELR1, KHSRP, KIAA2013, LAMP1, LYSMD3, MLLT1, MOSPD1, NSL1, NUCKS1, PFKFB3, PPCS, SLC25A44, ST6GALNAC6, TATDN3, TBCEL, THBS2, TNFRSF19, TSEN15, TSEN34, UBE2Q2, WDR26, WSB1, ZNF674, ZNF678	Gámez-Pozo[2], Merry[4]
	ATAD1, CPNE2, CSDE1, EFTUD2, EIF4ENIF1, SCUBE3, TIAM1, TMEM167A	Gámez-Pozo[2], Perez[9]
	ALDOA, ARHGAP18, ARL6IP5, CLINT1, ERICH1, FAM98A, FBXL19, FRYL, GRB10, HBS1L, ING3, ITIH5, MANBAL, MORF4L1, MTMR1, PHF21A, RBMS2, RPAIN, RRAS2, SHKBP1, TMEM120B, UTP15	Gámez-Pozo[2], Sorokin[8]
	PHB, RBP1, SH3BGRL	Khoury[1], Merry[4]
	NETO2, NXPH1, SLC12A2	Khoury[1], Sorokin[8]
	CDK5R1, ESR1, MEX3A, ORMDL3	Triulzi[3], Merry[4]
	VPS37B	Triulzi[3], Sorokin[8]
	CAMK2N1, CYP4X1, GATM	Guarneri[7], Merry[4]
	CNTNAP2, FAM84B	Guarneri[7], Khoury[1]
	BOC	Harris[6], Sorokin[8]

	SOX11	Triulzi[3], Zhao[5]
	ACTR1B	Gámez-Pozo[2], Sorokin[8], Perez[9]
	ARPC1A, FASTK, TXN	Gámez-Pozo[2], Sorokin[8], Merry[4]
	TOB1	Khoury[1], Sorokin[8], Merry[4]
	SFRP1	Khoury[1], Merry[4], Harris[6]
	RNF19A	Sorokin[8], Perez[9], Guarneri[7]
lincRNA differentially expressed in breast cancer tissues of cases compared to controls	AMPD2, BIRC6, C20orf202, CCDC68, CEP290, CILP, CLVS1, COL5A2, CSNK1A1, DDX51, DR1, EFR3B, EVI2A, EXD1, GMDS, HPGDS, IL1A, IQCJ, LOXL1, MAGI3, MCL1, MICB, NAALADL2, PDIA6, PEX3, PKMYT1, PRSS22, RFX3, RHOU, RHOXF2, RORA, SLC45A4, SPRED1, STX17, SUPT3H, TMED5, TRIM36, UBE2A, UTRN, VCAN, WIPF3, ZIC5, ZNF582, ZNF814	Merry[4]
CNA in breast cancer tissues of cases compared to controls	ABCB10, ABR, ABRA, ACBD3, ACTA1, ACTN2, ADORA1, AGT, AHCTF1, AKT3, ANGEL2, ANGPT1, ARID4B, ATAD2, ATF3, ATP2B4, ATP6V1C1, B3GALNT2, B3GALT2, BAALC, BROX, BTG2, C1orf116, C1orf198, C1orf21, C1orf74, C4BPA, CACYBP, CAMSAP2, CD34, CD46, CDC42BPA, CDC73, CDH4, CENPF, CHIT1, CHMP7, CHRM3, CNST, CNTN2, COL22A1, CR1, CR2, CSMD3, CSRP1, CTHRC1, DCAF13, DCAF4L2, DEGS1, DEPTOR, DERL1, DISC1, DNAH14, DSTYK, DUSP10, EBAG9, EDEM3, EDN3, EFCAB2, EGLN1, ELK4, ENAH, ENPP2, ENY2, EPRS, ESRRG, ETNK2, EXO1, EXOC8, FAM129A, FAM83A, FAM84B, FAM91A1, FBXO28, FBXO32, FBXO43, FLVCR1, FMOD, FZD6, GALNT2, GATA4, GGPS1, GNPAT, GPATCH2, GPR137B, GPR52, GREM2, GRHL2, HAS2, HHAT, HMCN1, HNRNPU, HSD11B1, IBA57, IGFN1, IKBKE, IPO9, IRF2BP2, IRF6, ITPKB,	Guarneri[7]

	IVNS1ABP, KCNH1, KCNK1, KCNK2, KCNT2, KCNV1, KCTD3, KDM5B, KIAA0040, KIF14, KIF21B, KIF26B, KLF10, KLHL20, LAD1, LAMC1, LAMC2, LAPTM4B, LEFTY2, LGALS8, LGR6, LIN9, LMX1A, LPGAT1, LRP12, LRRN2, LYPLAL1, LYST, MAL2, MAPKAPK2, MARK1, MDM4, MGST3, MIXL1, MMP16, MRPL13, MRPS14, MTDH, MTR, MTSS1, NAV1, NCALD, NCF2, NEK2, NEK7, NENF, NFASC, NID1, NIPAL2, NMNAT2, NR5A2, NSL1, NSMCE2, NUCKS1, NUP133, OPN3, OSR2, OTUD7B, OXR1, PBX1, PFKFB2, PGBD5, PHLDA3, PIGR, PIK3C2B, PKHD1L1, PKP1, PLA2G4A, PLD5, PLXDC1, PLXNA2, POLR2K, PPFIA4, PPP1R12B, PPP1R15B, PPP2R5A, PRG4, PROX1, PTDSS1, PTGS2, RAB22A, RAB3GAP2, RAB4A, RABGAP1L, RABIF, RAD21, RARA, RBBP5, RC3H1, RCOR3, RD3, RGL1, RGS2, RGS22, RHOU, RIMS2, RNF139, RNF19A, RNF2, RRM2B, RRP15, RSPO2, SAMD12, SDC2, SERTAD4, SHISA4, SIPA1L2, SLC13A5, SLC25A32, SLC26A9, SLC30A1, SLC30A8, SLC35F3, SLC41A1, SLC45A3, SLC7A13, SMG7, SNX31, SPAG1, SQLE, SRGAP2, STK3, SV2A, SWT1, SYBU, SYT14, TAF1A, TAF2, TAF5L, TARBP1, TATDN3, TGFB2, TIMM17A, TMCO1, TMEM206, TMEM65, TMEM74, TNFRSF11B, TNN, TNNI1, TOMM20, TP53BP2, TPR, TRAF5, TRIB1, TRIM58, TRIM67, TRMT12, TRMT1L, TRPS1, TSEN15, TSNAX, TSPYL5, TTC13, UBR5, UCK2, USH2A, UTP23, VAPB, VASH2, VPS13B, WDR26, WWP1, XAF1, YOD1, YWHAE, YWHAZ, ZBTB37, ZC3H11A, ZFPM2, ZHX1, ZHX2, ZNF124, ZNF281, ZNF572, ZNF670, ZNF678, ZNF706, ZNF831	
Protein differentially expressed in blood of breast cancer cases compared to controls	LDHA, MASP1, DBF4B	Yang[13]

CNA: Copy number alteration; lincRNA: long intergenic noncoding RNA.

Supplementary Table 15. Long intergenic noncoding RNA (lincRNA) differentially expressed in breast cancer tissues of cases compared to controls

**lincRNA**	**Direction change in expression in resistant tumor**	**Reference**
linc-PDE10A-1, linc-ZFP64-5, linc-ARF6-1, linc-FCGR1B-7, linc-SAA2, linc-ZC3H12B-2, linc-TM4SF4-2, linc-DIO3-8, linc-C11orf66, linc-BDH1-3, linc-EFR3A-6,	Decreased (*n* = 33)	Merry[4]

linc-ABCA5-7, linc-PHF10, linc-FAR2-2, linc-MUC20-2, linc-COL28A1, linc-ARPP21-2, linc-AP2B1-2, linc-NADSYN1-1, linc-ARL4D-2, linc-C3orf79-4, linc-NCKAP1-1, linc-SYNPO2-2, linc-SLC39A10-10, linc-TMEM18-13, linc-TMED5, linc-B4GALNT4-1, linc-FAIM3, linc-EFR3A-7, linc-ACTL7A-10, linc-KCNMB2-5, linc-USP16-5, linc-STMN2-1		
linc-TXLNB-1, linc-VCAN, linc-TMEM194A, linc-RNF113A, linc-CBR1-1, linc-DBT-3, linc-VPS8-2, linc-EFR3B, linc-PDE8B-2, linc-CST7-1, linc-KLHL31-1, linc-HTR2A-3, linc-IL18, linc-CLRN2-1, linc-IQCJ, linc-C5orf38-5, linc-TTC15-9, linc-MIA3-1, linc-LAMA4-3, linc-PRPS1L1, linc-RNFT2-1, linc-C4orf45-3, linc-LOC389493-2, linc-METAP1-2, linc-FAM91A1-3, linc-MCL1, linc-SCLT1-6, linc-KCTD7-2, linc-RPP40, linc-TMEM35, linc-MEST-2, linc-FBXO16-2, linc-ZNF8-1, linc-GABRA5-5, linc-TRIM39-1, linc-CEP110-2, linc-PKIA-1, linc-GABRA5-6, linc-KATNAL1-1, linc-RSPH3, linc-FAM114A1-1, linc-PARK2-4, linc-NR3C2-3, linc-NUSAP1, linc-S100B-2, linc-OR5AU1, linc-MAP3K9-1, linc-MCCC2, linc-CREB1-1, linc-IL4R-2, linc-PRSS22, linc-PIK3C2G-2, linc-TOX3-3, linc-ZNF681-4, linc-MAPKAP1-3, linc-UTRN, linc-STIL-2, linc-KIAA1755-3, linc-SNTG2-3, linc-CXCR4-1, linc-FLRT2-1, linc-LOXL1, linc-CSMD1-1, linc-NBPF15-1, linc-AMPD2, linc-HSPG2, linc-C9orf80, linc-RFX3, linc-NDUFV3-1, linc-POLR3A-1, linc-RP9-2, linc-SPATA7-3, linc-TMEM5, linc-DHFRL1-4, linc-COG3-2, linc-LRRTM3-3, linc-MEGF6, linc-WIPF3, linc-OPN4-1, linc-KIAA0141, linc-ZCCHC13-2, linc-DEC1, linc-NAALADL2, linc-NBPF9-9, linc-SAFB-2, linc-MAGI3, linc-TRIML2-5, linc-ZNF814, linc-LRCH2-1, linc-CSNK1A1, linc-NRG2-1, linc-TMEM18-7, linc-MGAT1-1, linc-BIRC6, linc-PPIAL4A-4, linc-EVX2-8, linc-ZNF582, linc-NAP1L2-3, linc-RGPD8-2, linc-FAM84B-4, linc-ATF3-1, linc-MYOT-4, linc-F13A1-1, linc-AGA-2, linc-SH3BGRL2-1, linc-GABRA5-7, linc-RHOXF1-2, linc-BRD9-1, linc-LPHN3-8, linc-ZNF8-2, linc-ZBED3, linc-MICB, linc-RNASEH2B-1, linc-RAPH1-2, linc-ZIC5, linc-DNAH8-2, linc-SUPT3H, linc-HIATL1-1, linc-PKMYT1, linc-MAP2K3-2, linc-PXMP4-2, linc-CCT8-2, linc-EXD1, linc-C5orf32-1, linc-TRIM29-3, linc-DR1, linc-SEPP1-1, linc-IDH3B-1, linc-SPRED1, linc-CSTB-1, linc-SLC25A48-2, linc-TPBG-3, linc-SEP15-3, linc-MRPL46, linc-PRKACB-2, linc-KLF6-2, linc-ZPBP2, linc-ALDH1B1-3, linc-ARHGAP28-2, linc-ARID3B-1, linc-DTL-2, linc-SRP9-1, linc-UBE2A, linc-SLC45A4, linc-STIM2-4, linc-PCF11-2, linc-NOP14-1, linc-ITGB2-1, linc-ANKRD56-1, linc-VIPR2-4, linc-ID2-3, linc-IRX3-5, linc-FAAH2-2, linc-ART1-1, linc-RBKS-1, linc-SLC6A19-1, linc-SSTR1-3, linc-PRKAA2-1, linc-METTL10, linc-CILP, linc-COL5A2, linc-CA5A-2, linc-YIPF5-3, linc-ABI1-1, linc-JAKMIP3-3, linc-SSTR1-2, linc-ZNF99-7, linc-ZKSCAN1-2, linc-LYSMD3-2, linc-WDR26-3, linc-KCNN4, linc-ZNF485-2, linc-ZNF227, linc-KIN-6, linc-FBXW4-3, linc-NBPF9-5, linc-CASP10-2, linc-IARS2-3, linc-OTUD7B-1, linc-	Increased (n = 338)	Merry[4]

GPATCH2-1, linc-ODF3B, linc-C10orf93-1, linc-IGSF5-1, linc-GARS-3, linc-MCTP2-4, linc-POLE4, linc-KLHL18-3, linc-WRB-2, linc-ESRRB-2, linc-SGCG-4, linc-PDCD1-1, linc-PCM1, linc-POLR3G-10, linc-EVI2A, linc-COMMD6-2, linc-RC3H1-2, linc-GPR65-6, linc-TGM3-1, linc-ZNF33A-5, linc-PLA2G2C-1, linc-C10orf122-2, linc-CCNH-2, linc-TRIM36, linc-RBMX-1, linc-C7orf23, linc-CD180-7, linc-C10orf107-1, linc-MAP1LC3B-2, linc-FOXA1-1, linc-CROCC-3, linc-ATF3-2, linc-MIB2, linc-AKAP9-1, linc-ADRA2A-1, linc-RORA, linc-CLASP2-1, linc-URB2-3, linc-OR2D2, linc-FAM71F2-3, linc-USP3-1, linc-CDH11-7, linc-SH3RF2, linc-GAD1-1, linc-DDX51, linc-ALOX12-1, linc-C5orf39, linc-SLC6A19-2, linc-CENPW-2, linc-BCL2A1-3, linc-CCDC39-1, linc-RNF2-1, linc-CCDC68, linc-USP12-5, linc-C6orf145-3, linc-CDH11-4, linc-HNRNPA1-3, linc-C20orf202, linc-PCDH8-4, linc-BTBD3-3, linc-RHOB-8, linc-GRIP1-3, linc-ARHGAP28-9, linc-FAM5C-4, linc-TTC15-1, linc-IRF2BP2-1, linc-ARFIP1-8, linc-GALM-1, linc-SPACA3, linc-NUDC, linc-ZNF648-2, linc-ZNF33A-4, linc-IL4R-1, linc-RNASEH1-12, linc-SHB, linc-ZNF99-5, linc-VN1R5-3, linc-ADRA2A-2, linc-GMDS, linc-NPSR1, linc-C1orf227-1, linc-MBOAT1-2, linc-AUTS2-5, linc-CCM2-2, linc-IKZF2-1, linc-BCL2A1-1, linc-FRG1-5, linc-CELF2-5, linc-MTRNR2L7, linc-GCA-1, linc-C2orf69-1, linc-RHOXF2, linc-HEATR6-2, linc-POLR3G-8, linc-RHOU, linc-RC3H1-3, linc-ZNF492-1, linc-DTNB, linc-BET1-1, linc-ASAP2-4, linc-ZNF726-3, linc-CPO-1, linc-CDH11-2, linc-S100G, linc-RAB40A-1, linc-DACT2-3, linc-EIF2AK3-1, linc-SATB2-2, linc-HERC6-2, linc-SP140-3, linc-CLIC2-2, linc-CIDEA-3, linc-IL1A, linc-STEAP1-3, linc-KIAA1024, linc-WDR53-1, linc-VPS8-3, linc-LRRC49-4, linc-RFPL4A, linc-RALGPS2-2, linc-USP24-2, linc-FOXB2-3, linc-ZNF131-3, linc-KLHDC7B, linc-TSHZ3-6, linc-GRAMD4-2, linc-PDIA6, linc-PHF21A-2, linc-MRGPRF-1, linc-MACF1-2, linc-LOC285033-4, linc-DUSP26-1, linc-RHOB-5, linc-NUDT6-1, linc-GUSB-5, linc-PEX3, linc-C18orf21, linc-CLVS1, linc-DOPEY1-2, linc-C2orf27A-2, linc-OPLAH, linc-RBKS-5, linc-TMEM99-2, linc-CDK13-2, linc-CEP290, linc-ZNF726-2, linc-APLN-1, linc-C9orf131, linc-SLC22A15-1, linc-CBR1-3, linc-CELF2-6, linc-NUF2-1, linc-HPGDS, linc-CPEB2-15, linc-STX17, linc-RTTN-5		

lincRNA: Long intergenic noncoding RNA.

Supplementary Table 16. Copy number alterations (CNA) in breast cancer tissues of cases compared to controls

Chromosome	Gene	Direction change in resistant	Reference

		**tumors compared to controls**	
Chr1	HIST2H2BE, BOLA1, SV2A, SF3B4, MTMR11, OTUD7B, VPS45, PBX1, LMX1A, RXRG, LOC400794, LRRC52, MGST3, ALDH9A1, TMCO1, UCK2, KLHL20, CENPL, DARS2, ZBTB37, SERPINC1, RC3H1, GPR52, RABGAP1L, CACYBP, MRPS14, TNN, KIAA0040, TNR, RFWD2, PAPPA2, NPL, DHX9, SHCBP1L, LAMC1, LAMC2, NMNAT2, SMG7, NCF2, ARPC5, APOBEC4, RGL1, GLT25D2, TSEN15, C1orf21, EDEM3, FAM129A, RNF2, TRMT1L, SWT1, IVNS1ABP, HMCN1, PRG4, TPR, C1orf27, OCLM, PDC, PTGS2, PLA2G4A, FAM5C, LOC440704, RGS18, RGS1, RGS13, RGS2, UCHL5, TROVE2, GLRX2, B3GALT2, CDC73, KCNT2, NEK7, ATP6V1G3, PTPRC, NR5A2, ZNF281, KIF14, DDX59, CAMSAP2, GPR25, KIF21B, CACNA1S, TMEM9, IGFN1, PKP1, TNNT2, LAD1, TNNI1, PHLDA3, CSRP1, NAV1, IPO9, SHISA4, LMOD1, TIMM17A, RNPEP, ELF3, GPR37L1, ARL8A, PTPN7, LGR6, UBE2T, PPP1R12B, SYT2, KDM5B, LOC148709, RABIF, KLHL12, ADIPOR1, CYB5R1, PPFIA4, MYOG, ADORA1, MYBPH, CHI3L1, CHIT1, LOC730227, BTG2, FMOD, PRELP, OPTC, ATP2B4, LAX1, ZBED6, ZC3H11A, SNRPE, SOX13, ETNK2, REN, KISS1, GOLT1A, PLEKHA6, LOC127841, PPP1R15B, PIK3C2B, MDM4, LRRN2, NFASC, CNTN2, RBBP5, DSTYK, TMCC2, NUAK2, KLHDC8A, LEMD1, CDK18, LOC284578, MFSD4, ELK4, SLC45A3, NUCKS1, RAB7L1, SLC41A1, PM20D1, SLC26A9, AVPR1B, C1orf186, CTSE, SRGAP2, IKBKE, RASSF5, EIF2D, DYRK3, MAPKAPK2, IL10, IL19, IL20, IL24, FAIM3, PIGR, FCAMR, C1orf116, YOD1, PFKFB2, C4BPB, C4BPA, CD55, CR2, CR1, CR1L, CD46, MIR29C, LOC148696, CD34, PLXNA2, LOC642587, MIR205, CAMK1G, LAMB3, G0S2, HSD11B1, TRAF3IP3, C1orf74, IRF6, DIEXF, SYT14, C1orf133, SERTAD4, HHAT, KCNH1, RCOR3, TRAF5, LINC00467, RD3, SLC30A1, NEK2, LPGAT1, INTS7, DTL, PPP2R5A, TMEM206, NENF, ATF3, FAM71A, BATF3, NSL1, TATDN3, C1orf227, FLVCR1-AS1, FLVCR1, VASH2, ANGEL2, RPS6KC1, PROX1, SMYD2, PTPN14, CENPF, KCNK2, KCTD3, USH2A, ESRRG, GPATCH2, SPATA17, RRP15, TGFB2, LYPLAL1, SLC30A10, EPRS, BPNT1, IARS2, RAB3GAP2, MARK1, C1orf115,	CN gain	Guarneri[7]

	MOSC2, MOSC1, HLX, DUSP10, HHIPL2, TAF1A, MIA3, BROX, DISP1, TLR5, SUSD4, C1orf65, CAPN8, CAPN2, TP53BP2, FBXO28, DEGS1, NVL, CNIH4, WDR26, CNIH3, DNAH14, LBR, ENAH, SRP9, EPHX1, TMEM63A, LEFTY1, PYCR2, LEFTY2, C1orf55, ACBD3, MIXL1, LIN9, PARP1, C1orf95, ITPKB, PSEN2, ADCK3, CDC42BPA, ZNF678, JMJD4, SNAP47, ARF1, C1orf35, MRPL55, GUK1, GJC2, IBA57, OBSCN, TRIM11, TRIM17, HIST3H3, RNF187, RHOU, RAB4A, SPHAR, C1orf96, ACTA1, NUP133, ABCB10, TAF5L, URB2, GALNT2, PGBD5, COG2, AGT, CAPN9, C1orf198, TTC13, ARV1, FAM89A, TRIM67, C1orf131, GNPAT, EXOC8, C1orf124, EGLN1, TSNAX, LOC100287814, DISC1, SIPA1L2, KIAA1383, NTPCR, PCNXL2, KIAA1804, KCNK1, SLC35F3, C1orf31, TARBP1, IRF2BP2, LINC00184, TOMM20, RBM34, ARID4B, GGPS1, TBCE, B3GALNT2, GNG4, LYST, NID1, GPR137B, ERO1LB, EDARADD, LGALS8-AS1, LGALS8, HEATR1, ACTN2, MTR, RYR2, ZP4, LOC100130331, LOC339535, CHRM3, FMN2, GREM2, RGS7, FH, KMO, OPN3, CHML, WDR64, EXO1, MAP1LC3C, PLD5, SDCCAG8, AKT3, ZNF238, C1orf100, ADSS, C1orf101, PPPDE1, FAM36A, HNRNPU-AS1, HNRNPU, EFCAB2, KIF26B, SMYD3, TFB2M, CNST, SCCPDH, LOC149134, AHCTF1, ZNF695, ZNF670, ZNF669, ZNF124, VN1R5, ZNF496, NLRP3, LOC148824, OR2C3, C1orf150, OR1C1, TRIM58, OR2L1P, OR2L13, OR2L2, OR2M4, LOC646627, SH3BP5L, ZNF672, ZNF692, PGBD2		
Chr8	PSKH2, ATP6V0D2, SLC7A13, WWP1, FAM82B, CPNE3, DCAF4L2, MMP16, UQCRB, MTERFD1, PTDSS1, SDC2, PGCP, TSPYL5, MTDH, LAPTM4B, MATN2, SNORA72, RPL30, C8orf47, HRSP12, POP1, NIPAL2, KCNS2, STK3, OSR2, VPS13B, COX6C, RGS22, FBXO43, POLR2K, SPAG1, RNF19A, ANKRD46, SNX31, YWHAZ, ZNF706, NACAP1, GRHL2, NCALD, RRM2B, UBR5, ODF1, KLF10, AZIN1, ATP6V1C1, C8orf56, BAALC, FZD6, CTHRC1, SLC25A32, DCAF13, RIMS2, TM7SF4, DPYS, LRP12, ZFPM2, OXR1, ABRA, ANGPT1, RSPO2, EIF3E, TTC35, TMEM74, TRHR, NUDCD1, ENY2, PKHD1L1, EBAG9, SYBU, KCNV1, CSMD3, TRPS1, EIF3H, UTP23, RAD21, RAD21-AS1, SLC30A8, MED30, EXT1, SAMD12, TNFRSF11B, COLEC10, MAL2, NOV, ENPP2, TAF2, DSCC1, DEPTOR, COL14A1, MRPL13, MTBP, SNTB1, HAS2, ZHX2, DERL1, WDR67, FAM83A, C8orf76, ZHX1,	CN gain	Guarneri[7]

	ATAD2, WDYHV1, FBXO32, ANXA13, FAM91A1, TMEM65, TRMT12, RNF139, NDUFB9, MTSS1, LOC157381, ZNF572, SQLE, KIAA0196, NSMCE2, TRIB1, FAM84B, MYC, PVT1, GSDMC, FAM49BKHDRBS3, COL22A1		
Chr17	LOC100131347, PLXDC1, RARA, GJD3	CN gain	Guarneri[7]
Chr20	C20orf85, PPP4R1L, RAB22A, VAPB, APCDD1L, LOC149773, STX16, ZNF831, EDN3, CDH4	CN gain	Guarneri[7]
Chr8	SGK223, GATA4, TNFRSF10A, LOC389641, CHMP7, ADAM5P	CN loss	Guarneri[7]
Chr17	ABR, TUSC5, YWHAE, SLC13A5, XAF1, FBXO39, STX8, WDR16	CN loss	Guarneri[7]
Overall	NR	CN burden higher<br>CN gain	Walsh[12]
Chr8	NR	CN burden higher	Walsh[12]
Chr17	NR	CN burden higher	Walsh[12]

Chr: Chromosome; CN: copy number; NR: not reported.

Supplementary Table 17. Protein differentially expressed in blood of breast cancer cases compared to controls

**Gene**	**Direction change in expression in resistant tumor**	**Referen ce**
APOA4, LDHB, LDHA, ADH5, TPT1	Decreased (n = 5)	Yang[13]
APOB, ITIH4, C9, ITIH3, MASP1, CD14, LCP1, CST3, LBP, MASP2, SRGN, SLC3A2, DBF4B	Increased (n = 13)	Yang[13]

Supplementary Table 18. Germline and somatic gene mutations in breast cancer tissues of cases compared to controls

**Gene**	**Type of mutations**	**Direction change in resistant tumor**	**Reference**
PIK3CA	Somatic	More mutations	Shi[14]
-	Germline	-	Lesurf[15]
-	Somatic	-	Lesurf[15]

References

1. Khoury T, Kanehira K, Wang D *et al.* Breast carcinoma with amplified HER2: a gene expression signature specific for trastuzumab resistance and poor prognosis. *Modern Pathology: An Official Journal of the United States and Canadian Academy of Pathology, Inc* 2010;23:1364-78. [DOI: 10.1038/modpathol.2010.125]

2. Gámez-Pozo A, Pérez Carrión RM, Manso L *et al.* The Long-HER study: clinical and molecular analysis of patients with HER2+ advanced breast cancer who become long-term survivors with trastuzumab-based therapy. *PloS One* 2014;9:e109611. [DOI: 10.1371/journal.pone.0109611]

3. Triulzi T, De Cecco L, Sandri M *et al.* Whole-transcriptome analysis links trastuzumab sensitivity of breast tumors to both HER2 dependence and immune cell infiltration. *Oncotarget* 2015;6:28173-82. [DOI: 10.18632/oncotarget.4405]

4. Merry CR, McMahon S, Forrest ME *et al.* Transcriptome-wide identification of mRNAs and lincRNAs associated with trastuzumab-resistance in HER2-positive breast cancer. *Oncotarget* 2016;7:53230-44. [DOI: 10.18632/oncotarget.10637]

5. Zhao B, Zhao Y, Sun Y *et al.* Alterations in mRNA profiles of trastuzumab-resistant Her-2-positive breast cancer. *Molecular Medicine Reports* 2018;18:139-46. [DOI: 10.3892/mmr.2018.8981]

6. Harris LN, You F, Schnitt SJ *et al.* Predictors of resistance to preoperative trastuzumab and vinorelbine for HER2-positive early breast cancer. *Clinical Cancer Research: An Official Journal of the American Association for Cancer Research* 2007;13:1198-207. [DOI: 10.1158/1078-0432.CCR-06-1304]

7. Guarneri V, Dieci MV, Frassoldati A *et al.* Prospective Biomarker Analysis of the Randomized CHER-LOB Study Evaluating the Dual Anti-HER2 Treatment With Trastuzumab and Lapatinib Plus Chemotherapy as Neoadjuvant Therapy for HER2-Positive Breast Cancer. *The Oncologist* 2015;20:1001-10. [DOI: 10.1634/theoncologist.2015-0138]

8. Sorokin M, Ignatev K, Barbara V *et al.* Molecular Pathway Activation Markers Are Associated with Efficacy of Trastuzumab Therapy in Metastatic HER2-Positive Breast Cancer Better than Individual Gene Expression Levels. *Biochemistry Biokhimiia* 2020;85:758-72. [DOI: 10.1134/S0006297920070044]

9. Perez EA, Thompson EA, Ballman KV *et al.* Genomic analysis reveals that immune function genes are strongly linked to clinical outcome in the North Central Cancer Treatment Group n9831 Adjuvant

Trastuzumab Trial. *Journal of Clinical Oncology: Official Journal of the American Society of Clinical Oncology* 2015;33:701-8. [DOI: 10.1200/JCO.2014.57.6298]
10. Du F, Yuan P, Zhao ZT *et al.* A miRNA-based signature predicts development of disease recurrence in HER2 positive breast cancer after adjuvant trastuzumab-based treatment. *Scientific Reports* 2016;6:33825. [DOI: 10.1038/srep33825]
11. Ohzawa H, Miki A, Teratani T *et al.* Usefulness of miRNA profiles for predicting pathological responses to neoadjuvant chemotherapy in patients with human epidermal growth factor receptor 2-positive breast cancer. *Oncology Letters* 2017;13:1731-40. [DOI: 10.3892/ol.2017.5628]
12. Walsh N, Andrieu C, O'Donovan P *et al.* Whole-exome sequencing of long-term, never relapse exceptional responders of trastuzumab-treated HER2+ metastatic breast cancer. *British Journal of Cancer* 2020;123:1219-22. [DOI: 10.1038/s41416-020-0999-z]
13. Yang T, Fu Z, Zhang Y, Wang M, Mao C, Ge W. Serum proteomics analysis of candidate predictive biomarker panel for the diagnosis of trastuzumab-based therapy resistant breast cancer. *Biomedicine & Pharmacotherapy = Biomedecine & Pharmacotherapie* 2020;129:110465. [DOI: 10.1016/j.biopha.2020.110465]
14. Shi W, Jiang T, Nuciforo P *et al.* Pathway level alterations rather than mutations in single genes predict response to HER2-targeted therapies in the neo-ALTTO trial. *Annals of Oncology: Official Journal of the European Society for Medical Oncology* 2019;30:1018. [DOI: 10.1093/annonc/mdy530]
15. Lesurf R, Griffith OL, Griffith M *et al.* Genomic characterization of HER2-positive breast cancer and response to neoadjuvant trastuzumab and chemotherapy-results from the ACOSOG Z1041 (Alliance) trial. *Annals of Oncology: Official Journal of the European Society for Medical Oncology* 2017;28:1070-7. [DOI: 10.1093/annonc/mdx048]
